# Compositionally Tunable Interpolymer System for Charge-Selective Recovery of Gold Cyanide from Ferrocyanide-Rich Solutions

**DOI:** 10.3390/polym18162016

**Published:** 2026-08-20

**Authors:** Meruyert Suleimenova, Talkybek Jumadilov, Juozas Gražulevičius, Khuangul Khimersen, Meruyert Mukanova

**Affiliations:** 1 Bekturov Institute of Chemical Sciences, 106 Sh. Ualikhanov Street, Almaty 050010, Kazakhstan; 2 Faculty of Natural Sciences and Geography, Abai Kazakh National Pedagogical University, 13 Dostyk Ave., Almaty 050010, Kazakhstan; 3 Department of Polymer Chemistry and Technology, Kaunas University of Technology, 73 K. Donelaicio Street, 44249 Kaunas, Lithuania

**Keywords:** interpolymer systems, mixed-bed ion exchangers, gold cyanide recovery, hexacyanoferrate(II) selectivity, pseudo-second-order kinetics, sulfonated polystyrene–divinylbenzene, hydrometallurgical processing

## Abstract

Selective recovery of gold from cyanide leach liquors is hindered by the co-dissolution of iron minerals that generate ferrocyanide complexes which strongly compete with [Au(CN)_2_]^−^ at ion-exchange sorbents. Here, we investigate mixed-bed interpolymer systems (IPS) composed of a strong-acid sulfonated polystyrene–divinylbenzene cation exchanger (TC007, Na^+^ form) and a strong-base quaternary ammonium anion exchanger (AV-17-8, Cl^−^ form) as a charge-selective platform for gold cyanide recovery. IPS compositions spanning cation-to-anion molar ratios from 6:0 to 0:6 were evaluated in batch contact with binary model solutions containing 30 mg L^−1^ each of [Au(CN)_2_]^−^ and [Fe(CN)_6_]^4−^ at pH 10 and 25 °C. The optimal 1:5 IPS achieved an [Au(CN)_2_]^−^ extraction degree of 79.88% and a selectivity coefficient β = D_Au_/D_Fe_ = 4.95 at 48 h, whereas the pure AV-17-8 anion exchanger (0:6) reached only 37.55% Au extraction at 48 h, following an atypical delayed-uptake kinetic profile rather than the rapid, near-quantitative capture expected of an unmodified strong-base resin. Sorption kinetics were best described by a pseudo-second-order model (R^2^ = 0.9992), confirming ion exchange at quaternary ammonium sites as the dominant rate-controlling step, with a ~30-fold increase in k_2_ for [Au(CN)_2_]^−^ in the 1:5 IPS relative to AV-17-8 alone. FTIR spectroscopy and TGA-DSC revealed the incorporation of metal cyanide complexes into the IPS matrix, with diagnostic C≡N stretching bands at 2108.7 and 2034.1 cm^−1^ and an additional thermal event at 200–280 °C. These findings establish compositionally tunable IPS based on commercially available resins as a charge-selective sorbent platform demonstrating a capacity to regenerate under single-cycle elution conditions for gold cyanide recovery from ferrocyanide-containing process streams while highlighting the need for further evaluation under industrial Fe:Au ratios.

## 1. Introduction

Gold remains one of the most strategically important precious metals, underpinning financial reserves, electronics manufacturing, and medical applications globally [1,2,3]. Cyanide leaching is the dominant industrial method for extracting gold from ores, accounting for over 90% of primary gold production worldwide [4,5]. However, the selectivity of downstream recovery processes is severely compromised by the co-dissolution of iron-containing minerals, which generates ferrocyanide complexes—predominantly hexacyanoferrate(II), [Fe(CN)_6_]^4−^—that compete directly with the target dicyanoaurate(I) anion, [Au(CN)_2_]^−^, during sorption-based recovery [6]. The presence of ferrocyanide at concentrations that can exceed those of gold by one to two orders of magnitude in process liquors represents one of the most persistent challenges in hydrometallurgical gold recovery [7]. In typical cyanide leach liquors from pyrite-bearing gold ores, the [Fe(CN)_6_]^4−^:[Au(CN)_2_]^−^ molar ratio routinely reaches 50:1 to 200:1 [8], imposing a severe thermodynamic penalty on anion-exchange selectivity, as conventional sorbents cannot discriminate between the target anion and a vastly more abundant competitor of similar chemical character. This leads to reduced sorbent loading capacity, accelerated fouling, and substantial economic losses. While selective gold sorption using a KU-2-8/AV-17-8 IPS was previously demonstrated [9], the present study employs TC007 (Lanlang^®^), a gel-type sulfonated PS-DVB resin with a higher nominal capacity (≥1.9 meq/mL) and a different bead size distribution, to assess whether the selectivity phenomenon is reproducible across alternative cation-exchanger partners and to provide a more complete kinetic and spectroscopic characterization absent from the prior study. A preliminary communication by Jumadilov et al. [10] reported qualitative gold sorption from iron-rich solutions using a dual-phase IPS; the present study extends that work through systematic compositional optimization across the full 6:0–0:6 molar ratio range, full kinetic modeling (PFO, PSO, and Weber–Morris), and FTIR/TGA-DSC mechanistic characterization of the loaded sorbent matrix.

Activated carbon in carbon-in-pulp (CIP) and carbon-in-leach (CIL) circuits remains the industrial benchmark for [Au(CN)_2_]^−^ recovery [11] but exhibits limited selectivity toward [Au(CN)_2_]^−^ over [Fe(CN)_6_]^4−^, requires high-temperature regeneration, and suffers from organic fouling and attrition [12]. Strong-base quaternary ammonium resins on polystyrene–divinylbenzene (PS–DVB) matrices exhibit high affinity for gold cyanide complexes via classical anion exchange, yet their selectivity collapses in ferrocyanide-rich liquors where anions of similar charge and hydration compete at high ionic strength [13,14,15,16,17,18,19,20]. Recent studies on functionalized and chelating resins have achieved improved gold–iron selectivity but at the cost of synthesis complexity and challenging regeneration, constraining their industrial deployment.

Interpolymer systems [21,22,23,24,25,26] represent a conceptually distinct class of sorption materials, formed by the physical combination of cation-exchange and anion-exchange polymers in a single mixed-bed configuration. IPS, first systematically developed by the Kazakhstani school of polymer chemistry led by Prof. Jumadilov, exploits synergistic electrostatic interactions at the interface between cation- and anion-exchange polymers. The resulting biphasic microenvironment generates localized electric field gradients that impose differential constraints on ions of differing charge density and molecular geometry. Critically, the strong negative electrostatic field of the sulfonate groups (–SO_3_^−^) in the cation-exchange component is predicted to impose greater repulsion on the highly charged [Fe(CN)_6_]^4−^ (charge: −4) than on the singly charged [Au(CN)_2_]^−^ (charge: −1), providing a thermodynamic basis for size- and charge-selective discrimination. The exclusion of the more highly charged [Fe(CN)_6_]^4−^ from the vicinity of the fixed sulfonate charge can be qualitatively rationalized by Donnan potential theory, in which the equilibrium distribution of a mobile ion of charge z between a charged gel phase and bulk solution follows exp(−zFΨ_D/RT_), where Ψ_D_ is the Donnan potential established by the fixed sulfonate charge density. Because [Fe(CN)_6_]^4−^ carries four times the charge of [Au(CN)_2_]^−^, its exclusion is expected to be strongly favored at a given Ψ_D_, though the magnitude of this effect depends on ionic strength, hydration, and steric factors not captured by this simplified treatment and requires further validation (e.g., by streaming-potential measurements or Poisson–Boltzmann modeling of the interfacial region). Despite the established theoretical framework for IPS in water treatment and analytical chemistry applications, their application to the selective recovery of gold cyanide complexes from ferrocyanide-containing hydrometallurgical streams has not been systematically investigated.

The composition of IPS (specifically the molar ratio of the cation-exchange to anion-exchange component) is expected to be a decisive variable governing both overall sorption capacity and ion selectivity. Yet no prior study has rigorously optimized this parameter for the [Au(CN)_2_]^−^/[Fe(CN)_6_]^4−^ binary system, nor has the kinetic and mechanistic basis of selectivity in such mixed-exchanger systems been elucidated using modern kinetic modeling and spectroscopic methods. Furthermore, the application of commercially available, industrially scalable ion exchangers—such as sulfonated PS-DVB resins in the Na^+^ form paired with strong-base quaternary ammonium resins in the Cl^−^ form—offers a practically viable route for IPS deployment without requiring custom synthesis.

The present work represents the first systematic investigation of compositionally tunable interpolymer systems as a platform for charge-selective separation of dicyanoaurate(I) from hexacyanoferrate(II) in binary hydrometallurgical model solutions. While IPS materials have been studied in water treatment and analytical chemistry contexts, no prior study has (i) optimized the cation-to-anion exchanger molar ratio specifically for the [Au(CN)_2_]^−^/[Fe(CN)_6_]^4−^ system, (ii) rigorously elucidated the kinetic mechanism of selectivity using modern PSO, PFO, and Weber–Morris models across the full compositional range, or (iii) provided molecular spectroscopic and thermal evidence (FTIR, TGA-DSC) for the mode of complex fixation within the IPS matrix. The conceptual advance introduced here—the use of a cation-exchange component not as an active sorption phase but as a charge-selective interfacial barrier exploiting Born electrostatic repulsion scaling with the square of ionic charge within the IPS microenvironment, sulfonate groups (–SO_3_^−^) in the cation-exchange phase generate a residual anionic field that is expected to repel highly charged anions such as [Fe(CN)_6_]^4−^ more strongly than singly charged [Au(CN)_2_]^−^, providing a thermodynamic basis for charge-dependent discrimination. Qualitative Donnan and Born electrostatic considerations suggest that repulsion increases approximately with the square of ionic charge; however, the magnitude of this effect in the present system likely depends on ionic strength, hydration, and steric constraints and has not yet been quantified by electrokinetic measurements or continuum modeling. Accordingly, we treat this charge-selective barrier concept as a working mechanistic hypothesis that is evaluated indirectly through kinetic, selectivity, and spectroscopic data. Unlike conventional strong-base anion exchangers (e.g., Purolite A500, Lewatit MonoPlus M500), which achieve near-quantitative extraction of all anionic species without charge-density discrimination (β = 1), and unlike gold-specific chelating resins that require custom synthesis and complex regeneration, the proposed IPS achieves β = 4.95 using commercially available, mechanically mixed industrial-grade resins regenerable by 9% thiourea + 2% H_2_SO_4_ elution—a practically significant combination of selectivity, cost, and operational simplicity not previously demonstrated for this separation. Furthermore, the approximately 30-fold enhancement in PSO rate constant observed for the optimal 1:5 IPS (k_2_ = 0.8348 g mg^−1^ h^−1^) relative to the pure anion exchanger (k_2_ = 0.0273 g mg^−1^ h^−1^)—attributed to mutual activation of functional groups at the cation/anion exchanger interface—represents an additional and previously unreported kinetic benefit of the IPS architecture that has not been observed or rationalized in prior sorption studies.

Building on recent demonstrations of IPS-based selective gold sorption in KU-2-8/AV-17-8 and PMAA–P4VP systems, the present work systematically interrogates the composition–selectivity relationship in a mixed-bed IPS prepared from commercially available TC007(Na^+^) and AV-17-8(Cl^−^) resins. Specifically, we (i) optimize the cation-to-anion exchanger molar ratio for the [Au(CN)_2_]^−^/[Fe(CN)_6_]^4−^ binary, (ii) apply modern PFO, PSO, and Weber–Morris kinetic analyses across the full 6:0–0:6 composition range, and (iii) provide FTIR and TGA-DSC evidence for metal cyanide complex incorporation into the IPS matrix. We further explore the concept of a cation-exchange phase acting as a charge-selective interfacial barrier rather than an active sorption phase, and evaluate its practical consequences for selectivity, kinetics, and ability to regenerate.

## 2. Materials and Methods

### 2.1. Ion-Exchange Materials

Two commercially available ion-exchange resins were used in this study. The cation-exchange component was Lanlang^®^ TC007 (Taiyuan Lanlang Technology Industrial Corp., Taiyuan, China), a gel-type strong-acid cation-exchange resin based on a sulfonated polystyrene–divinylbenzene (PS-DVB) copolymer matrix, supplied in the Na^+^ ionic form. The key physicochemical properties of TC007 are as follows: total ion-exchange capacity ≥ 1.9 meq/mL, moisture content 45–50%, effective bead diameter 0.4–0.6 mm, bulk density 0.77–0.87 g/mL, specific gravity 1.25–1.29 g/mL, and uniformity coefficient < 1.7. The resin is thermally stable up to 120 °C and is operable across the full pH range (0–14).

**Figure 1:**
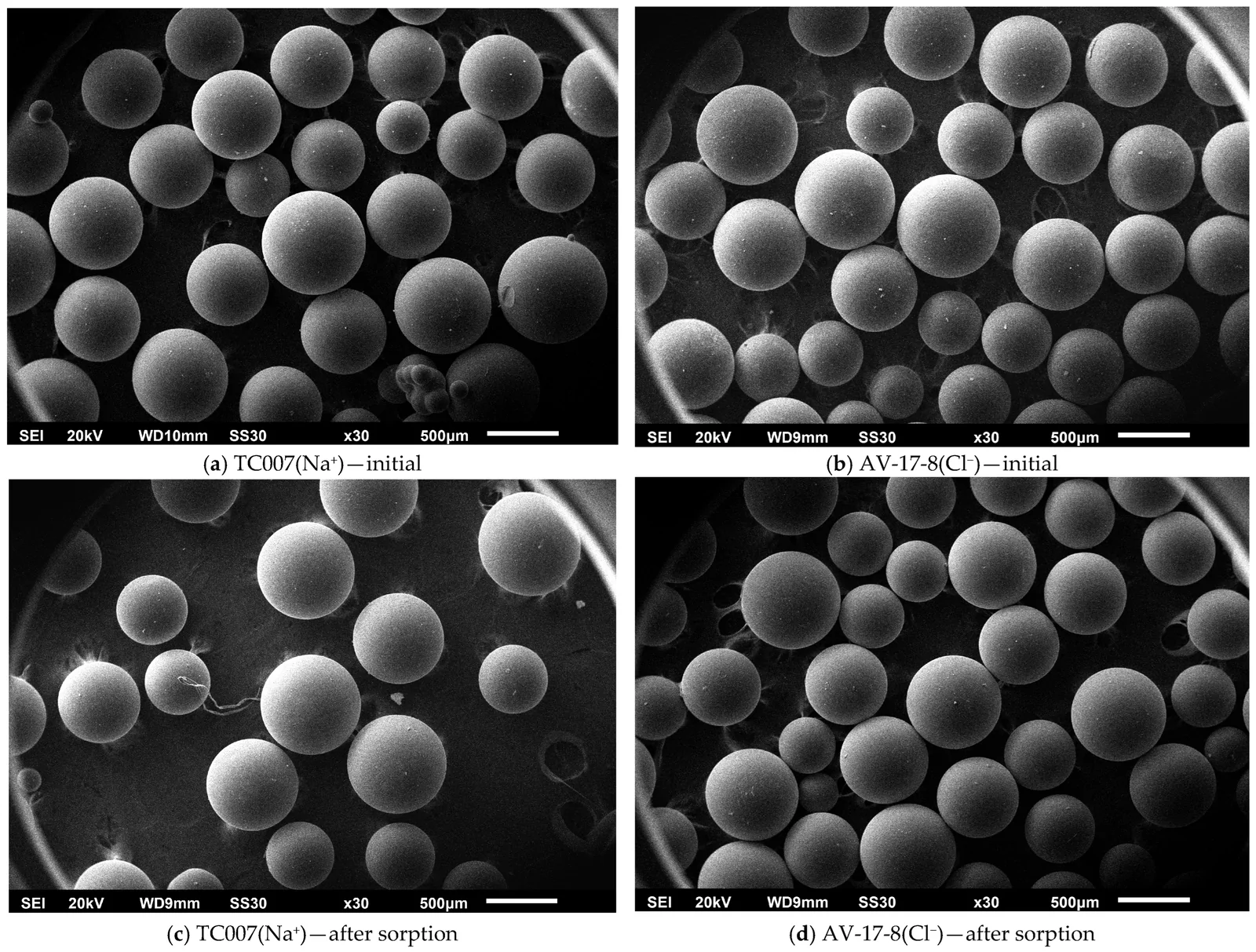
SEM micrographs (×30 magnification, 500 µm scale) of (**a**) TC007(Na^+^) before sorption and (**b**) AV-17-8(Cl^−^) before sorption, (**c**) TC007(Na^+^) after sorption, and (**d**) AV-17-8(Cl^−^) after sorption.

The anion-exchange component was AV-17-8(Cl^−^ form), a strong-base gel-type anion-exchange resin bearing quaternary ammonium functional groups on a PS-DVB matrix. Prior to use, both resins were conditioned by sequential washing with distilled water, 9% thiourea + 2% H_2_SO_4_ solution, and distilled water again until the conductivity of the effluent stabilized, followed by air-drying to constant mass under ambient conditions. The morphology of the ion-exchange beads was examined both by optical microscopy at 30× magnification and by SEM. Figure 1 presents representative SEM micrographs of TC007(Na^+^) and AV-17-8(Cl^−^) in their initial state and after sorption, revealing the spherical bead geometry and surface characteristics of each resin.

The key physicochemical properties of AV-17-8(Cl^−^) are: total exchange capacity ≥ 0.9 meq/mL (manufacturer nominal, wet-volume basis), moisture content 43–48%, effective bead diameter 0.3–1.2 mm, and a gel-type (non-macroporous) crosslinked polystyrene–divinylbenzene matrix consistent with Type I strong-base quaternary ammonium resins. Because both TC007 and AV-17-8 are gel-type, non-macroporous PS-DVB resins with comparable moisture content, the IPS studied here is best regarded as a compositionally tunable binary mixed-bed system in which the tunable variable is the equivalent ratio of fixed-charge sites, rather than an engineered interfacial architecture with independently controlled crosslinking density, porosity, or bead-scale contact geometry. Direct measurement of crosslinking degree, swelling ratio, and inter-resin contact characteristics was outside the scope of the present study and is identified as a priority for future mechanistic work (Section 4.6).

In this work, IPS composition is defined by the equivalent ratio of exchange sites (m_eq_ of TC007 vs. AV-17-8), rather than by mass or volume alone, ensuring that composition changes correspond to systematically varied densities of sulfonate and quaternary ammonium sites.

### 2.2. Preparation of Interpolymer Systems

Interpolymer systems (IPS) were prepared by mixing the pre-conditioned TC007(Na^+^) and AV-17-8(Cl^−^) resins in seven defined cation-to-anion molar ratios: 6:0, 5:1, 4:2, 3:3, 2:4, 1:5, and 0:6, where the ratio represents the equivalent proportion based on the ion-exchange capacity of each component. The total sorbent mass in each system was kept constant. Mixed systems were equilibrated by gentle agitation in deionized water for 24 h prior to sorption experiments to allow inter-resin electrostatic equilibration and eliminate any released ions from the exchanger surface.

Equivalent amounts of each resin were calculated from experimentally determined dry-mass exchange capacities rather than manufacturer-reported wet-volume values to eliminate ambiguity arising from moisture content variability. The total ion-exchange capacity of TC007(Na^+^ form) was determined by acid–base titration to be 4.41 ± 0.34 m_eq_/g (dry basis); the corresponding capacity of AV-17-8(Cl^−^ form) was determined to be 2.97 ± 0.17 m_eq_/g (dry basis). The moisture content of each resin, determined gravimetrically by drying to constant mass at 105 °C, was 47.5 ± 2.5% for TC007 and 43.0 ± 3.0% for AV-17-8. These dry-mass capacities and moisture contents were used to calculate the dry mass of each resin required for every IPS composition (Table 1), and it is this dry mass—not the wet mass at the time of mixing—that was used as the denominator m in Equation (2) for all reported q_t_, distribution coefficient (D), and kinetic constant values. Table 1 has been revised to report both the wet mass used for practical handling during IPS preparation and the corresponding dry mass used for all quantitative calculations.

### 2.3. Model Solutions

Binary model solutions were prepared by dissolving potassium dicyanoaurate(I), K[Au(CN)_2_], and potassium hexacyanoferrate(II), K_4_[Fe(CN)_6_]·3H_2_O (analytical grade, ≥99%), in deionized water (resistivity ≥ 18.2 MΩ·cm) to obtain initial concentrations of 30 mg/L for each metal ion. K[Au(CN)_2_] (≥98%, Sigma-Aldrich, Merck, St. Louis, MO, USA) and K_4_[Fe(CN)_6_]·3H_2_O (≥99%, Thermo Scientific Chemicals, Waltham, MA, USA) were used as received without further purification and stored desiccated at room temperature in the dark prior to use, consistent with the manufacturer’s recommendations for hydrolytically and photolytically sensitive cyanide salts. NaOH used for pH adjustment (0.1 M) was prepared from analytical-grade pellets (≥98%, Sigma-Aldrich, St. Louis, MO, USA). Because K_4_[Fe(CN)_6_] can undergo photodecomposition under light exposure [27], all sorption and desorption vessels (contact times up to 72 h) were wrapped in aluminum foil or kept in a dark cabinet for the duration of each experiment to minimize photolytic artifacts.

All solutions were prepared fresh prior to each experiment. The pH of each freshly prepared binary solution was adjusted to 10.0 ± 0.2 using 0.1 M NaOH and verified using a calibrated glass pH electrode. No buffer or additional supporting electrolyte was used, thereby avoiding the introduction of additional competing anions. The solution was mixed thoroughly after pH adjustment and used for sorption experiments on the day of preparation. Between preparation, sampling, and analysis, solutions were kept in sealed polyethylene vessels at ambient laboratory temperature to minimize evaporation, contamination, and changes in solution composition.

Batch experiments were performed in sealed polyethylene vessels containing 50.0 mL of the model solution. At each predetermined contact time, a 1.0 mL aliquot of the supernatant was withdrawn, filtered through a 0.45 µm membrane filter, and analyzed for residual Au and Fe by ICP-OES.

### 2.4. Batch Sorption Experiments

Batch sorption experiments were conducted by contacting a fixed mass (Table 1) of each IPS with 50 mL of the binary model solution in sealed polyethylene vessels under constant mechanical agitation (150 rpm) at 25 ± 1 °C. Samples of the supernatant solution (1.0 mL aliquots) were withdrawn at contact times of 0.5, 2.5, 6, 24, and 48 h, filtered through 0.45 µm membrane filters, and analyzed for residual Au and Fe concentrations. All experiments were performed in triplicate, and mean values are reported.

The sorption degree (E, %) was calculated according to Equation (1):

(1) $$E=\frac{C_{0}-C_{t}}{C_{0}}\times 100\%,$$

where C_0_ (mg/L) is the initial metal concentration and *Ct* (mg/L) is the residual concentration at time *t*.

The amount of metal sorbed per unit mass of sorbent, *q*_*t*_ (mg/g), was calculated according to Equation (2):

(2) $$q_{t}=\frac{{(C}_{0}-C_{t})V}{m},$$

where V (L) is the solution volume and m (g) is the dry mass of the sorbent.

The selectivity coefficient β was defined as the ratio of the distribution coefficients of gold and iron was calculated according to Equation (3):

(3) $$\beta =\frac{D_{Au}}{D_{Fe}},\;D=\frac{q_{e}}{C_{e}}$$

where *q*_*e*_ (mg/g) is the equilibrium sorption capacity and *C*_*e*_ (mg/L) is the equilibrium concentration in solution.

Statistical significance of differences in extraction degree among the seven IPS compositions was assessed at each contact time by one-way analysis of variance (ANOVA), followed by Tukey’s honestly significant difference (HSD) post-hoc test for pairwise comparisons, using a significance threshold of α = 0.05.

### 2.5. Desorption Experiments

Desorption kinetics were studied by transferring the metal-loaded sorbents from the 48-h sorption experiments into 50 mL of 9% thiourea + 2% H_2_SO_4_ solution as eluent. Aliquots were collected at contact times of 0.5, 2.5, 6, 24, 48, and 72 h and analyzed for desorbed Au and Fe concentrations by AES. Desorption efficiency (D, %) was calculated according to Equation (4):

(4) $$D_{des}=\frac{C_{des}\cdot V_{des}}{q_{e}\cdot m}\times 100\%,$$

where *C*_*des*_ (mg/L) is the metal concentration in the eluate and V_des_ (L) is the eluent volume.

Desorption behaviour was evaluated using 9% thiourea + 2% H_2_SO_4_ solution as a strongly complexing eluent for Au(I). For both eluents, metal-loaded sorbents from the 48 h sorption experiments were transferred into 50 mL of eluent and contacted for up to 72 h under the same agitation conditions; aliquots were analysed by ICP-OES to ensure consistent detection limits. Desorption degree (R, %) was defined by Equation (5):

(5) $$R=\frac{m_{desorbed}}{m_{sorbed}}\times 100\%,$$

where m_desorbed_ and m_sorbed_ are the cumulative masses of metal desorbed and initially sorbed onto the IPS, respectively, calculated from concentration–volume products.

### 2.6. Kinetic Modeling

Sorption kinetics were analyzed using three models. The pseudo-first-order (PFO) model [28]:

(6) $$\frac{{dq}_{t}}{dt}=k_{1}(q_{e}-q_{t}),$$

The pseudo-second-order (PSO) model [29]:

(7) $$\frac{t}{q_{t}}=\frac{1}{k_{2}q_{e}^{2}}+\frac{t}{q_{e}},$$

The Weber–Morris intraparticle diffusion model [30]:

(8) $$q_{t}=k_{id}\cdot t^{0.5}+C,$$

where *k*_1_ (1/h) and *k*_2_ (g/mg·h) are the rate constants of the PFO and PSO models, respectively; *q*_*e*_ (mg/g) is the calculated equilibrium sorption capacity; *k*_*i*d_ (mg/g·h^0.5^) is the intraparticle diffusion rate constant; and *C* is the intercept related to the boundary layer thickness.

Kinetic parameters were estimated by nonlinear regression of the untransformed q_t_-versus-t data using the Levenberg–Marquardt algorithm implemented in OriginPro 2024. All data points were assigned equal weighting, and convergence was accepted when the change in chi-square between successive iterations was below 10^−6^. The fitted parameters for the PFO and PSO models were the equilibrium sorption capacity, q_e_, and the respective rate constant, k_1_ or k_2_. Parameter estimates are reported with two-sided 95% confidence intervals calculated from the nonlinear-regression covariance matrix.

Model performance was assessed using the coefficient of determination (R^2^), adjusted coefficient of determination ($R_{adj}^{2}$), root-mean-square error (RMSE), reduced chi-square ($\chi_{v}^{2}$), and corrected Akaike information criterion (AICc). Lower RMSE, $\chi_{v}^{2}$, and AICc values were considered indicative of a better fit, whereas higher $R_{adj}^{2}$, values indicated a greater proportion of variance explained. Residuals were inspected for random distribution around zero and the absence of systematic time-dependent deviations. Because each kinetic profile contained five experimental time points, the confidence intervals and model-comparison metrics are interpreted as indicators of relative fit quality within the present data set rather than as independent mechanistic proof.

### 2.7. Equipment

The surface morphology and elemental composition of the samples were characterized using a JSM-6610LV scanning electron microscope (JEOL Ltd., Tokyo, Japan) equipped with an energy-dispersive X-ray spectroscopy system for microanalysis (Oxford Instruments, Abingdon, UK). Individual beads of TC007(Na^+^) and AV-17-8(Cl^−^) were mounted on aluminum stubs with double-sided conductive carbon tape, gently air-dried, and examined at a working distance of 9–10 mm and a nominal magnification of ×30. Images were acquired with a 500 µm scale bar to resolve bead morphology and surface texture before and after sorption from the binary Au(CN)_2_/Fe(CN)_6_. Residual ion concentrations were determined using an inductively coupled plasma optical emission spectrometer (ICP-OES, iCAP PRO XP, Thermo Fisher Scientific, Loughborough, Leicestershire, UK) across a wavelength range of 167.021–852.145 nm. Fourier Transform Infrared (FTIR) spectroscopy (transmission mode) (Nicolet 5700, Thermo Fisher Scientific, Madison, WI, USA) was utilized to investigate changes in functional groups, with samples prepared by mixing with potassium bromide (KBr) and pressing into pellets, acquiring spectra in the range of 500–4000 cm^−1^ at 0.4 cm^−1^ resolution with 32 scans. Simultaneous thermogravimetric and differential scanning calorimetry (TGA/DSC) analysis was performed using an SKZ1052 DSC thermal analyzer (SKZ Industrial Co., Ltd., Shanghai, China). Approximately 5–10 mg of each sample was placed in an aluminum crucible and heated from ambient temperature to 600 °C at a programmed heating rate of 10 °C/min under a dynamic nitrogen atmosphere (flow rate: 50 mL/min) to prevent oxidative degradation.

## 3. Results

### 3.1. Effect of Interpolymer System Composition on Sorption Efficiency

#### 3.1.1. Influence of Molar Ratio on Gold and Iron Extraction

**Figure 2:**
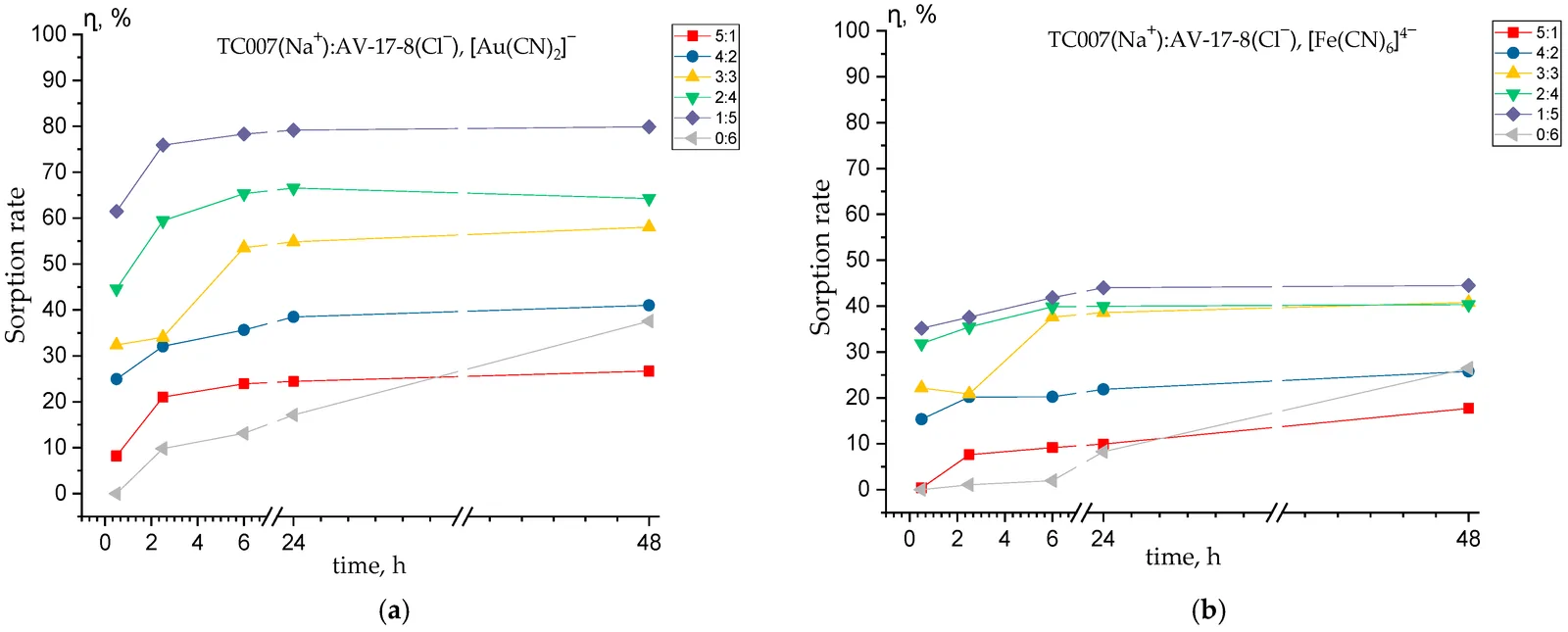
Time-dependent sorption degrees of [Au(CN)_2_]^−^ (**a**) and [Fe(CN)_6_]^4−^ (**b**) at different TC007(Na^+^):AV-17-8(Cl^−^) molar ratios (6:0–0:6) in binary solutions (30 mg L^−1^ each, pH 10, 25 °C). Error bars represent ±1 SD (*n* = 3). The 1:5 IPS exhibits the highest gold extraction and pronounced suppression of ferrocyanide uptake, revealing a non-monotonic composition–selectivity relationship with a maximum at intermediate cation-exchanger loading.

The effect of TC007(Na^+^):AV-17-8(Cl^−^) molar ratio on the extraction degree of [Au(CN)_2_]^−^ and [Fe(CN)_6_]^4−^ was investigated across seven compositions from 6:0 (pure cation exchanger) to 0:6 (pure anion exchanger) at an initial concentration of 30 mg/L for each ion. The results at all five contact times are summarized in Figure 2.

Several clear trends emerged from the data (Figure 2): At the 6:0 ratio (pure TC007, Na^+^ form), no sorption of either Au or Fe was observed at any contact time, confirming that the sulfonate cation exchanger repels both anionic cyanide complexes as expected; At the 0:6 ratio (pure AV-17-8, Cl^−^ form), Au extraction followed an unusual non-monotonic-rate profile: 0% at 0.5 h, rising slowly to 10.0% (2.5 h), 13.0% (6 h), and 17.0% (24 h), before accelerating sharply to 37.5% by 48 h. This late-stage acceleration increases 20.5 percentage points between 24 and 48 h, exceeding the cumulative increase over the preceding 24 h—is inconsistent with classical film- or particle-diffusion-limited ion exchange, in which the sorption rate is expected to decelerate monotonically as available sites are depleted (Section 3.2 and Section 3.3.5). At intermediate IPS compositions, the sorption degree of both ions increased progressively with increasing AV-17-8 content and with contact time. The 1:5 ratio exhibited the highest selective performance, achieving 79.88% Au extraction with a selectivity coefficient β = 4.95 relative to [Fe(CN)_6_]^4−^ at the defined equilibrium time point. One-way ANOVA confirmed that IPS composition had a statistically significant effect on Au extraction degree at 48 h (*p* < 0.05); Tukey’s post-hoc test showed that the 1:5 composition differed significantly from the 2:4 and 3:3 compositions (*p* < 0.05), confirming that the observed selectivity optimum is not an artifact of experimental variability.

The non-monotonic relationship between IPS composition and selectivity is particularly noteworthy. While the 3:3 ratio produced comparable extraction degrees for both ions (54.84% Au vs. 38.62% Fe at 24 h), the 2:4 and 1:5 ratios demonstrated progressively enhanced Au preference, with the 2:4 system achieving 66.54% Au vs. 39.98% Fe at 24 h. This behavior indicates that the cation-exchange component exerts a differential steric and electrostatic inhibitory effect that is disproportionately stronger toward the more highly charged [Fe(CN)_6_]^4−^ (charge: −4) than toward [Au(CN)_2_]^−^ (charge: −1).

#### 3.1.2. Temporal Evolution of Sorption and Equilibrium Attainment

The temporal profiles of Au and Fe extraction at the optimal 1:5 IPS ratio are shown in Figure 2. The sorption degree of gold increased rapidly from 61.45% at 30 min to 75.89% at 2.5 h, reached 78.32% at 6 h and 79.15% at 24 h, and stabilized at 79.88% by 48 h, indicating that the great majority of Au uptake (>75%) occurs within the first 30 min of contact, consistent with rapid access to readily available exchange sites at this optimal composition. The corresponding Fe extraction followed a similar trend but consistently lower values throughout the kinetic profile, confirming persistent Au preference across the entire course. The marginal change in sorption degree between 24 h and 48 h (less than 2% for both ions) was taken as the operational criterion for equilibrium attainment.

### 3.2. Sorption Kinetics

#### Pseudo-First-Order and Pseudo-Second-Order Models

Kinetic modeling was performed for the optimal 1:5 IPS ratio using the PFO, PSO, and Weber–Morris models. The nonlinear regression results showed that the PSO model provided a better description of the experimental kinetic data compared to the PFO model across all investigated IPS compositions, as evidenced by higher coefficients of determination (R^2^ = 0.7531–0.9992 for PSO vs. R^2^ = 0.5156–0.8499 for PFO) and lower chi-square values (χ^2^).

For the 1:5 system, the PSO-calculated equilibrium sorption capacities (*qe*, *calc*) were in close agreement with the experimentally determined values (*qe*, *exp*), further validating the model’s applicability. The PSO rate constant *k*_2_ for [Au(CN)_2_]^−^ was higher than that for [Fe(CN)_6_]^4−^ at the same IPS ratio, indicating that gold cyanide not only reaches higher equilibrium uptake but also exchanges at a faster intrinsic rate within the IPS matrix. This kinetic advantage further supports the suitability of the 1:5 IPS for selective gold recovery under time-constrained process conditions.

The dominance of the PSO model over the PFO model is mechanistically significant: PSO kinetics are consistent with chemisorption as the rate-determining step, which in the context of ion-exchange systems implies that the exchange of the target anion at the quaternary ammonium site (rather than external film diffusion) controls the overall rate of sorption.

### 3.3. Characterization of Ion-Exchange Materials

#### 3.3.1. SEM Characterization

SEM micrographs of TC007(Na^+^) and AV-17-8(Cl^−^) before and after sorption (Figure 1) show that both resins consist of nearly spherical beads with smooth surfaces and a narrow bead-size distribution. The initial TC007 beads appear slightly larger on average than AV-17-8, consistent with the manufacturer-reported effective diameters. No significant surface cracking or fragmentation is observed after contact with the binary cyanide solution, indicating that the IPS preparation and sorption conditions do not induce mechanical degradation of the PS–DVB matrices. The preservation of bead integrity after sorption supports the use of these commercially available resins as mechanically robust components in the mixed-bed IPS and confirms that the two phases remain morphologically distinct, which is important for interpreting composition-dependent sorption behavior.

**Figure 3:**
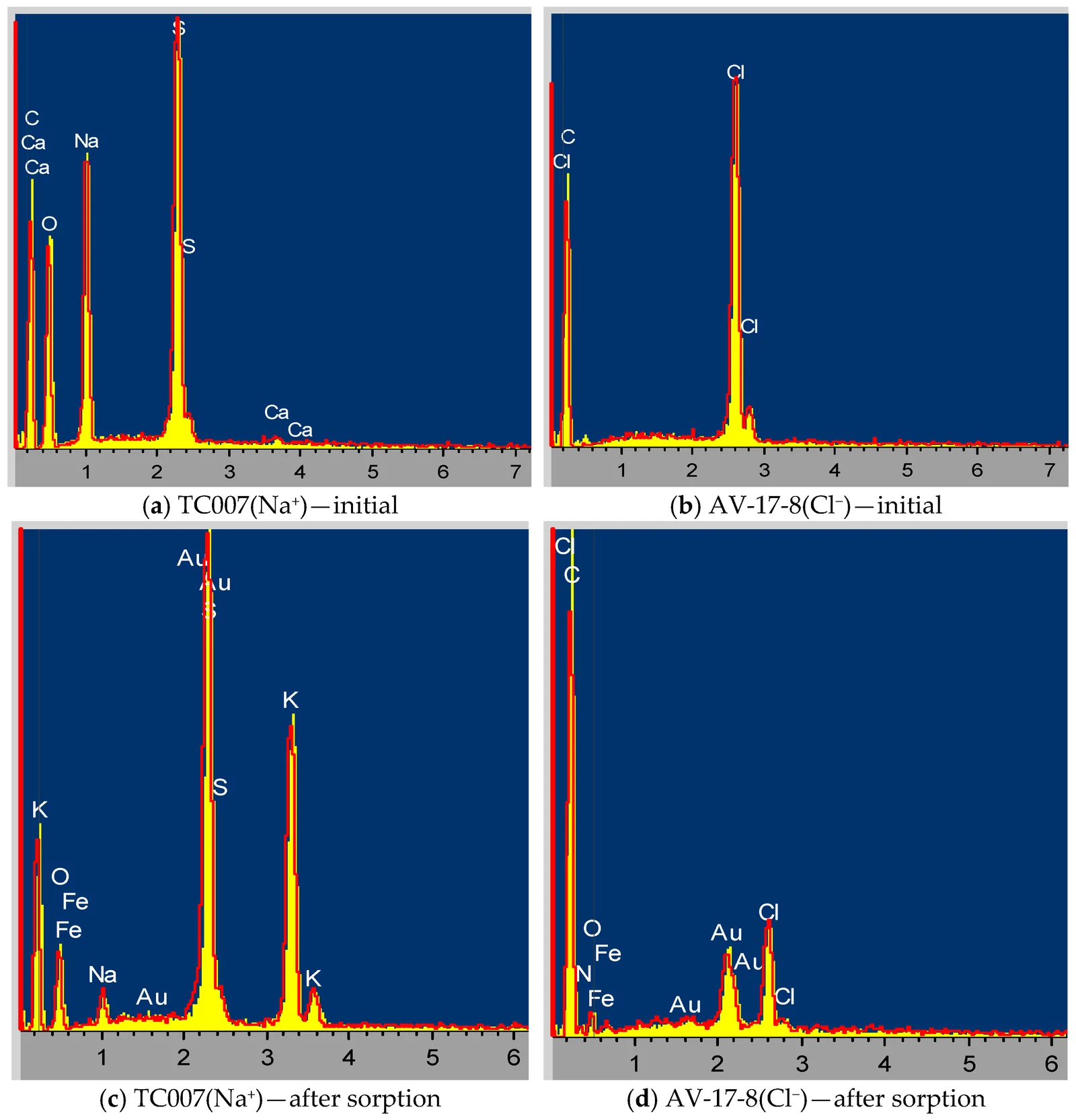
EDX spectra of (**a**) TC007(Na^+^) before sorption, (**b**) AV-17-8(Cl^−^) before sorption, (**c**) TC007 (Na^+^) after sorption, and (**d**) AV-17-8(Cl^−^) after sorption. Note the appearance of Au and Fe peaks only after sorption (**c**,**d**).

The elemental composition of the TC007(Na^+^) and AV-17-8(Cl^−^) before and after sorption obtained by SEM-EDX before and after contact with the binary [Au(CN)_2_]^−^/[Fe(CN)_6_]^4−^ solution provides direct evidence of Au, Fe uptake by both polymers (Table 2, Figure 3). The initial (unloaded) samples of TC007(Na^+^) and AV-17-8(Cl^−^) exhibited surface compositions dominated by C, O, and S, consistent with the sulfonated polystyrene–divinylbenzene matrix; no Au, Fe signals were detected in either unloaded resin (Figure 3a,b). After sorption, distinct Au and Fe peaks appeared in the EDX spectra of both resins (Figure 3c,d).

#### 3.3.2. FTIR Spectroscopic Characterization of the Interpolymer System TC007(Na^+^):AV-17-8(Cl^−^) (X:Y)

**Figure 4:**
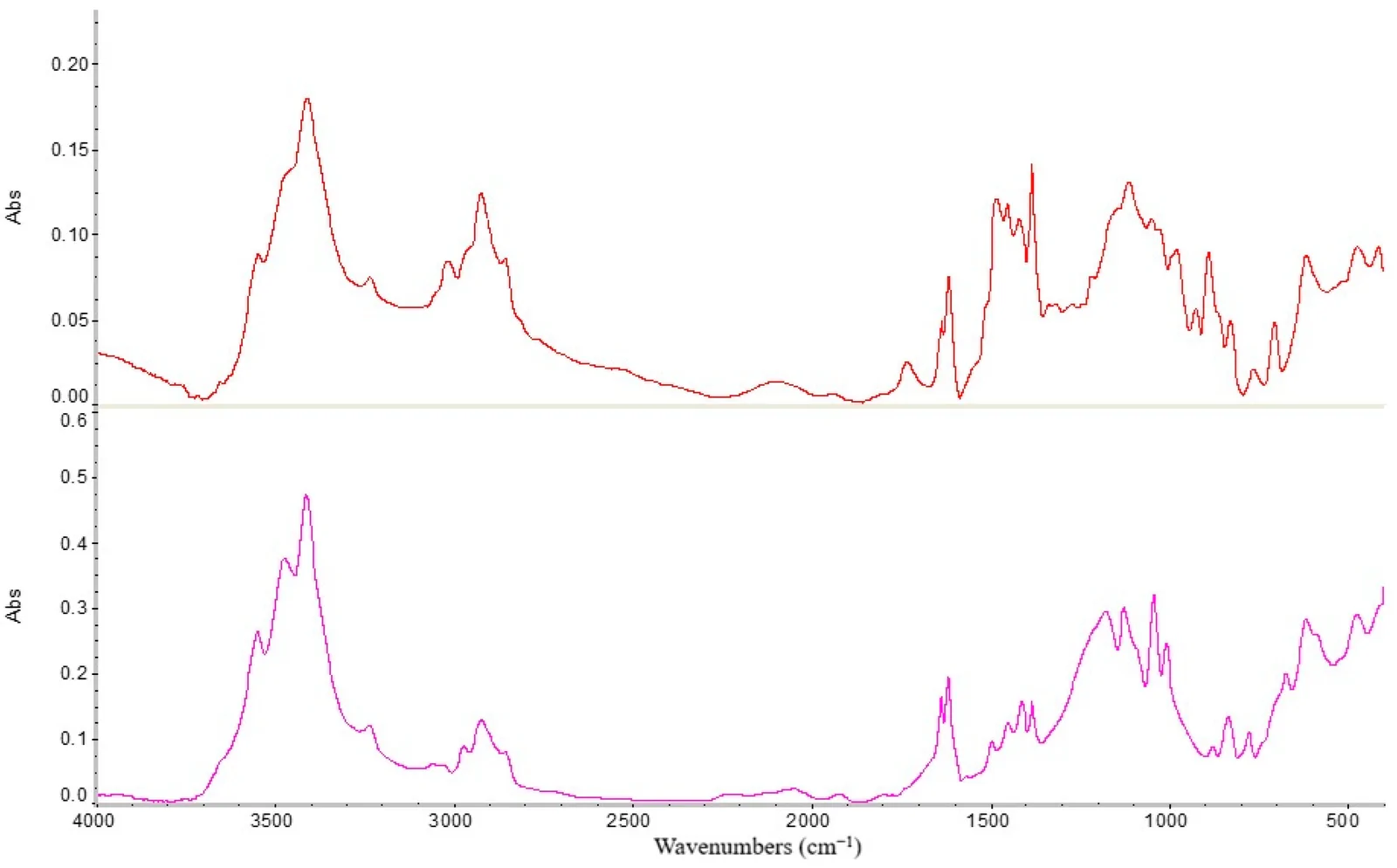
FTIR spectra of initial TC007(Na^+^) (red line), TC007 (Na^+^) from the interpolymer system TC007(Na^+^):AV-17-8(Cl^−^) (1:5) after sorption (purple line).

The FTIR spectra of the individual ion-exchange resins and their interpolymer systems were recorded before and after sorption to establish baseline spectral profiles and to identify sorption-induced structural changes. The spectrum of TC007(Na^+^ form) (Figure 4) in its initial state exhibited characteristic bands of the sulfonated PS-DVB matrix: aromatic C–H stretching vibrations at ~3030 cm^−1^, aliphatic C–H stretching at ~2920 cm^−1^, aromatic C=C ring vibrations at ~1600 and ~1490 cm^−1^, and strong asymmetric and symmetric S=O stretching bands of the sulfonate group (–SO_3_^−^) at ~1180 and ~1040 cm^−1^, respectively. No bands in the C≡N stretching region (1900–2200 cm^−1^) were observed in the initial spectrum, confirming the absence of cyanide species in the preconditioned resin.

**Figure 5:**
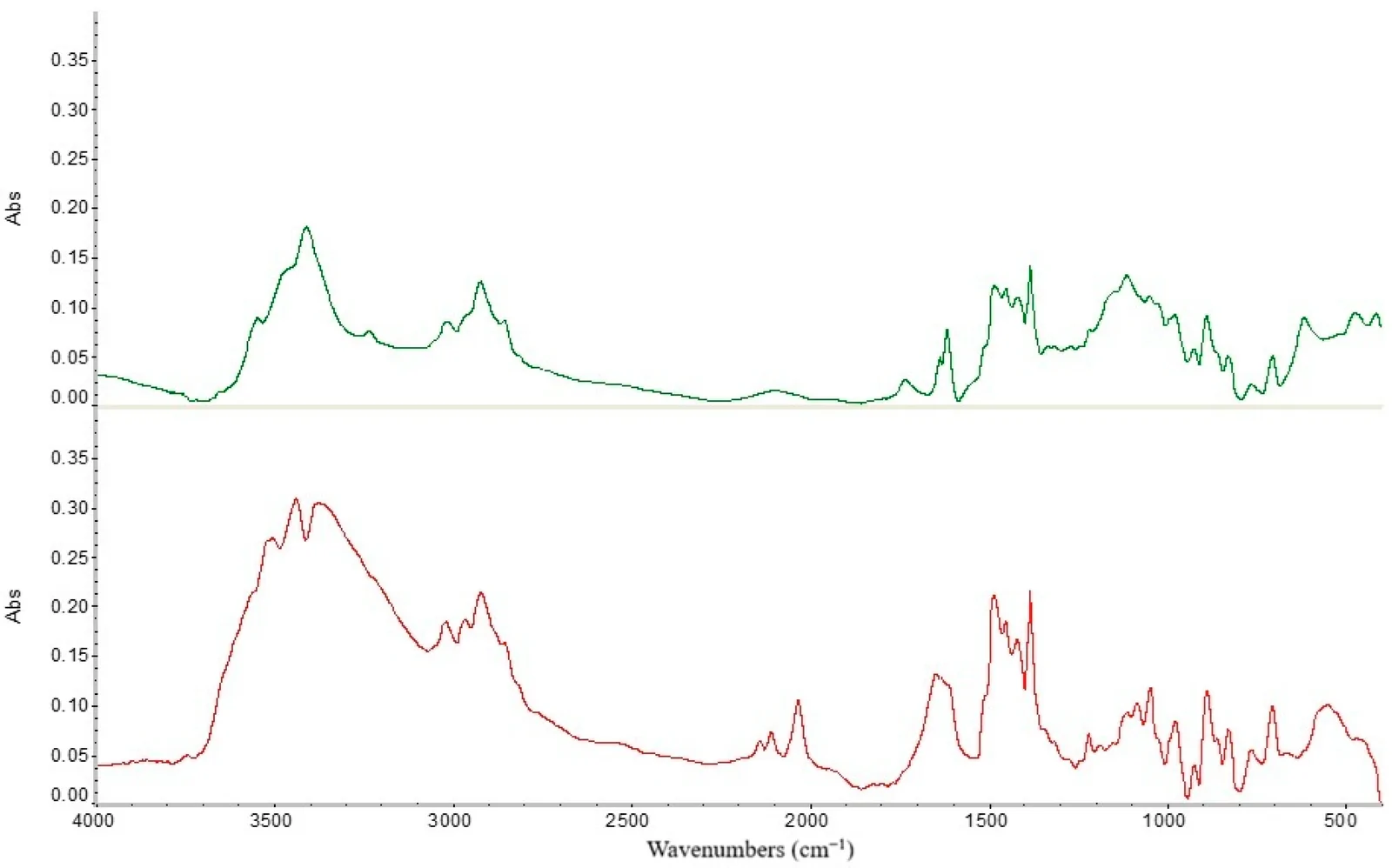
FTIR spectra of initial AV-17-8(Cl^−^) (green line), AV-17-8(Cl^−^) from interpolymer system TC007(Na^+^):AV-17-8(Cl^−^) (1:5) after sorption (red line).

The FTIR spectrum of AV-17-8(Cl^−^ form) (Figure 5) in its initial state displayed the characteristic PS-DVB polymer backbone bands, as well as the quaternary ammonium functional group signature: C–N stretching vibrations at ~960 cm^−1^ associated with the –N^+^(CH_3_)_3_ group, and N–CH_3_ deformation bands at ~1480 cm^−1^. These bands confirm the strong-base Type I quaternary ammonium character of the anion exchanger.

Following sorption from the binary [Au(CN)_2_]^−^/[Fe(CN)_6_]^4−^ solution, new absorption bands appeared in the C≡N stretching region of the post-sorption spectra of AV-17-8-containing systems. Specifically, two distinct bands were observed at 2108.7 cm^−1^ and 2034.1 cm^−1^, which are assigned to C≡N stretching vibrations of metal cyanide complexes fixed within the sorbent matrix. The band at 2108.7 cm^−1^ is attributed to the coordinated cyanide ligand of [Au(CN)_2_]^−^ retained within the anion-exchange matrix, while the band at 2034.1 cm^−1^ indicates a perturbation of the C≡N stretching frequency consistent with the restricted coordination environment experienced by the metal–cyanide complex upon ion exchange. The appearance of these bands confirms that both gold and iron cyanide complexes are chemically fixed within the IPS matrix via ion-exchange interactions rather than simple surface adsorption. Importantly, the C≡N bands were not observed in the post-sorption spectrum of the pure TC007(Na^+^) system (ratio 6:0), which is consistent with the electrostatic repulsion of anionic cyanide complexes by the negatively charged sulfonate groups and corroborates the zero-sorption observed for this system.

#### 3.3.3. Thermogravimetric and Differential Scanning Calorimetry Analysis

**Figure 6:**
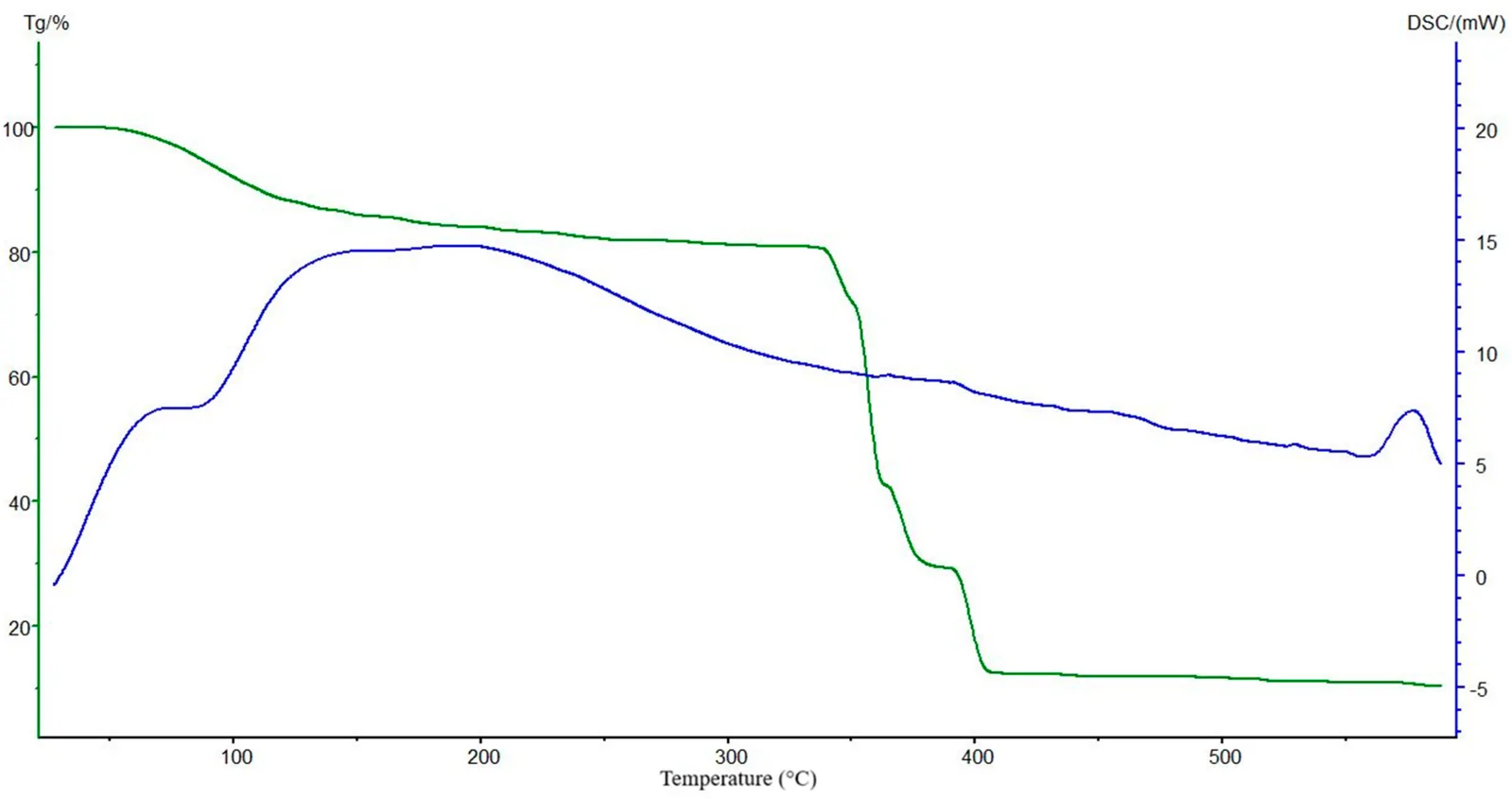
The TG (green line), and DSC (blue line) analyses of the initial TC007 (Na^+^). Legend: Mass loss (%) vs. temperature (°C); reduced mass loss up to 100 °C post-sorption indicates enhanced thermal stability due to metal coordination. Conditions: 10 °C/min heating rate under N_2_ flow.

**Figure 7:**
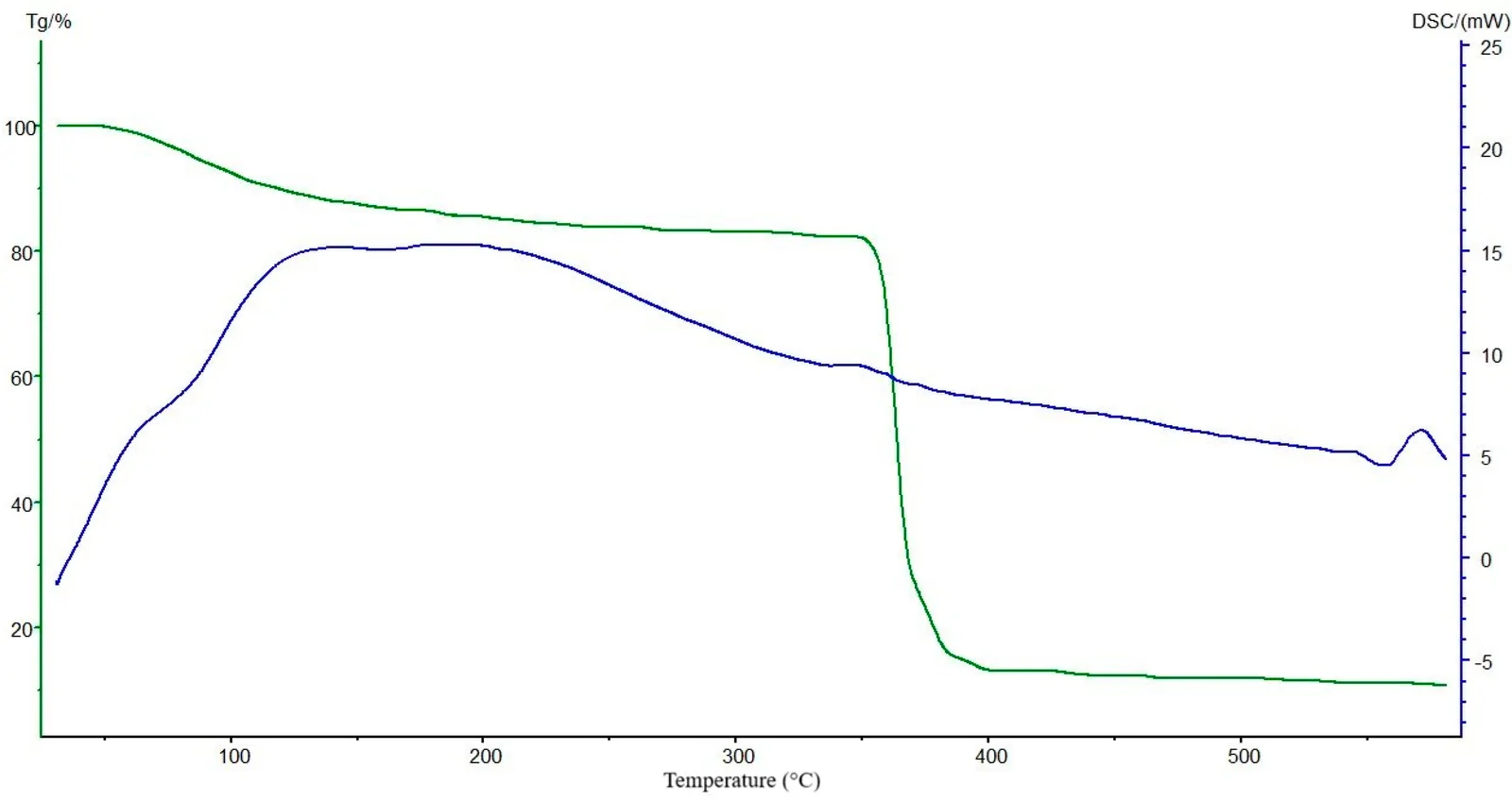
The TG (green line), and DSC (blue line) of TC007 (Na^+^) from the interpolymer system TC007(Na^+^):AV-17-8(Cl^−^) (1:5) after sorption. Legend: Mass loss (%) vs. temperature (°C). Conditions: 10 °C/min heating rate under N_2_ flow.

TGA-DSC analysis was performed on the individual resin (Figure 6) and selected IPS configurations (Figure 7) before and after sorption to evaluate thermal stability and to obtain indirect evidence of metal complex retention. The TGA curve of TC007(Na^+^) in its initial state showed a two-stage mass loss profile: a first stage below 150 °C corresponding to the release of physically adsorbed and interstitial water (mass loss: ~8–10%), and a second major decomposition stage in the range 280–420 °C associated with the thermal degradation of sulfonate functional groups and the PS-DVB polymer backbone. The DSC curve displayed a broad endothermic event below 150 °C (dehydration) and a sharp exothermic peak at ~320 °C corresponding to sulfonate decomposition.

**Figure 8:**
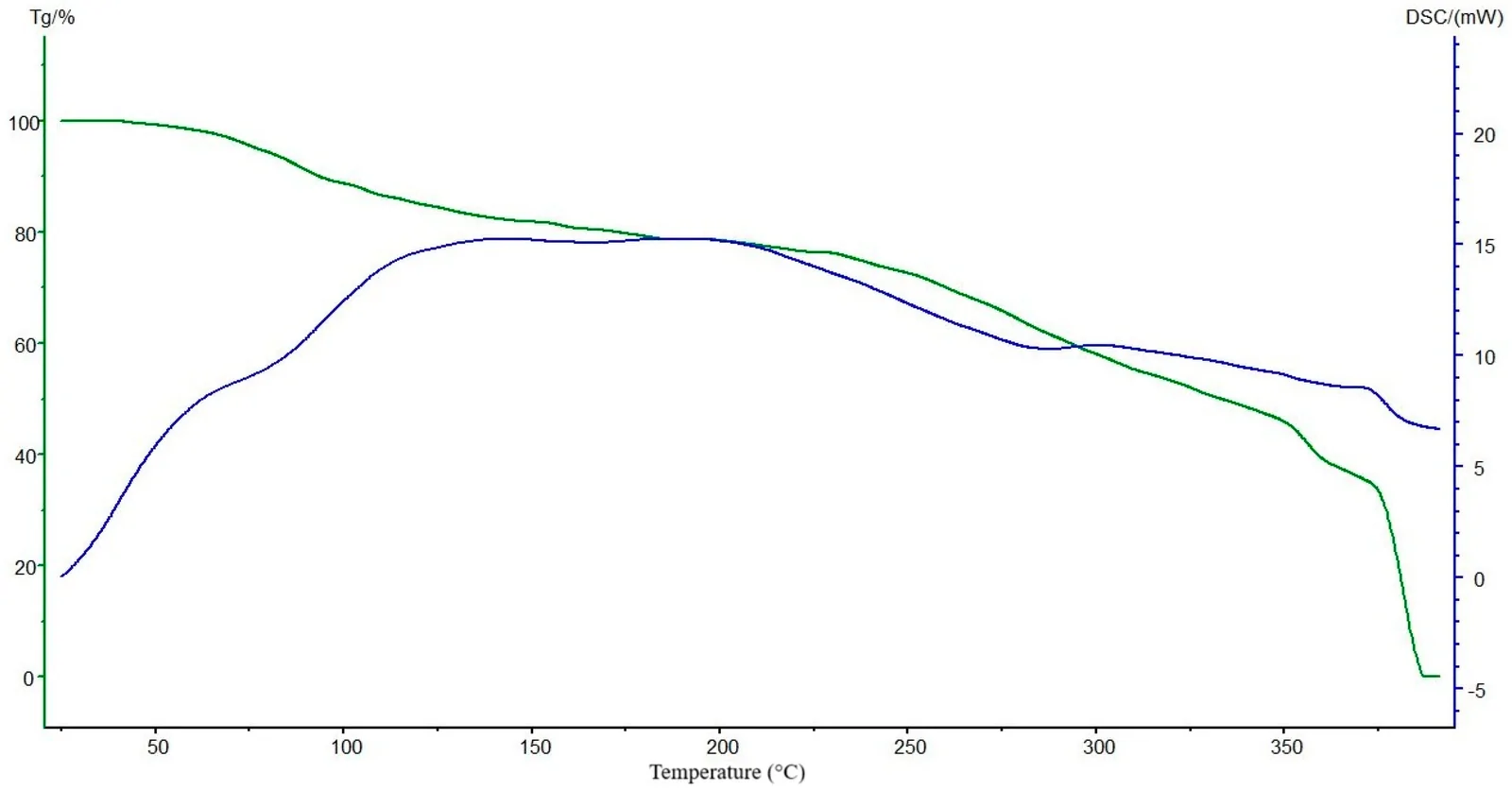
The TG (green line), and DSC (blue line) analyses of the initial AV-17-8(Cl^−^). Legend: Heat flow (mW/mg) vs. temperature (°C); endothermic peaks at ~80–120 °C shift post-sorption, reflecting stabilized polymer matrix. Conditions: 10 °C/min heating rate under N_2_ flow.

**Figure 9:**
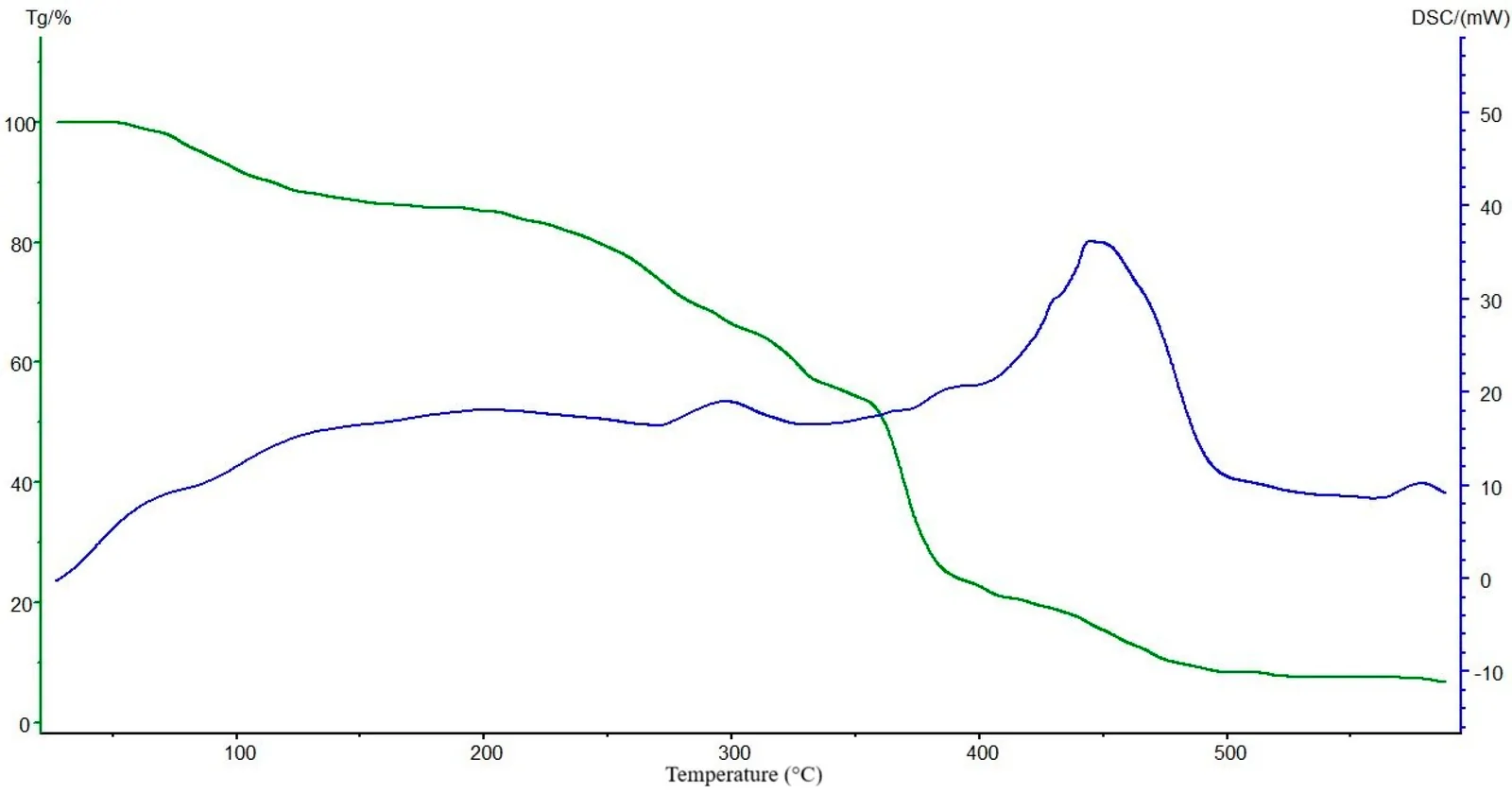
The TG (green line), DSC (blue line), and DTG (gray line) analyses of AV-17-8(Cl^−^) from the interpolymer system TC007(Na^+^):AV-17-8(Cl^−^) (1:5) after sorption. Legend: Heat flow (mW/mg) vs. temperature (°C); endothermic peaks at ~80–120 °C shift post-sorption, reflecting stabilized polymer matrix. Conditions: 10 °C/min heating rate under N_2_ flow.

Following sorption, the TGA curves of initial AV-17-8(Cl^−^) (Figure 8) and AV-17-8 (1:5)-containing IPS configurations (Figure 9) exhibited a modified decomposition profile relative to the pre-sorption baseline: a shift in the onset decomposition temperature and the appearance of an additional mass loss event in the 200–280 °C range, attributed to the decomposition of thermally retained metal–cyanide complexes within the polymer matrix. The DSC curves confirmed this interpretation through the appearance of a new endothermic feature in the same temperature range post-sorption, which was absent in the virgin resin. These findings are consistent with the FTIR evidence and support the conclusion that metal–cyanide complexes are genuinely incorporated into the IPS structure.

The comparative TGA-DSC analysis of multiple IPS configurations—including KU-2-8(Na^+^):AV-17-8(Cl^−^), Lewatit(Na^+^):AV-17-8(Cl^−^), Lewatit(H^+^):AV-17-8(Cl^−^), and TC007(Na^+^):AV-17-8(Cl^−^)—confirmed that the thermal response upon sorption is a general feature of anion-exchanger-containing interpolymer systems and is not specific to a single cation-exchange partner.

#### 3.3.4. Weber–Morris Intraparticle Diffusion Analysis

The Weber–Morris plots of *qt* versus *t*^0.5^ for the 1:5 IPS system exhibited multilinear profiles for both [Au(CN)_2_]^−^ and [Fe(CN)_6_]^4−^, indicating that the overall sorption process proceeds through more than one diffusion stage. Three linear segments were identified:

1. A steep initial segment (0–2.5 h) attributed to rapid external film diffusion and uptake at the most accessible surface sites of the AV-17-8 component;
2. An intermediate segment (2.5–24 h) with a reduced slope, representing intraparticle diffusion through the gel-phase PS-DVB matrix of the exchanger beads;
3. A final plateau segment (24–48 h) approaching equilibrium, where diffusion resistance becomes negligible.

#### 3.3.5. Comparative Kinetic Modeling and Identification of the Rate-Controlling Sorption Mechanism

As shown in Table 3, the PFO model showed relatively low correlation coefficients (R^2^ = 0.5156–0.8499), indicating a poorer fit to the experimental data compared with the other kinetic models considered. The Weber–Morris model revealed that intraparticle diffusion contributes to the sorption process. However, the deviation of the plots from the origin and the obtained R^2^ values (0.4920–0.9038) indicate that intraparticle diffusion is not the sole rate-controlling step. Therefore, the overall sorption process is governed by a combination of external diffusion, intraparticle diffusion, and interactions with active sorption sites.

For [Au(CN)_2_]^−^, the PSO rate constant increases from k_2_ = 0.0273 g mg^−1^ h^−1^ for the pure AV-17-8 resin (0:6) to k_2_ = 0.8348 g mg^−1^ h^−1^ at the 1:5 IPS, corresponding to ~30-fold acceleration of the apparent exchange rate. In contrast, ferrocyanide exhibits its highest k_2_ at 2:4 (k_2_ = 1.2483 g mg^−1^ h^−1^), reflecting complex composition-dependent interactions between diffusion and ion exchange for the tetra-charged species. Thus, kinetic enhancement is most pronounced for gold cyanide at the selectivity-optimal composition.

The poor PSO fit for the pure AV-17-8 system (R^2^ = 0.7531 for [Au(CN)_2_]^−^, Table 3; R^2^ = 0.0207 for [Fe(CN)_6_]^4−^, Table 4) is consistent with the non-monotonic-rate profile described in Section 3.1.1, in which Au and Fe uptake at 0:6 accelerate between 24 and 48 h rather than decelerating as classical PFO/PSO kinetics require. Both models assume a rate that is highest at early times and decreases monotonically as exchange sites are occupied; neither can accommodate a late-stage rate increase. We hypothesize that this behavior reflects a secondary, slower uptake process specific to the unmodified AV-17-8 resin—potentially involving reorganization of the resin’s hydrated gel structure or slow diffusion into less accessible internal exchange sites—that is either absent or kinetically outcompeted by the dominant, PSO-consistent ion-exchange process in all IPS compositions containing TC007. This anomaly is specific to the pure anion exchanger and does not affect the kinetic conclusions drawn for the selectivity-optimal 1:5 IPS, which shows excellent PSO agreement (R^2^ = 0.9992) and a conventional decelerating rate profile. Extended contact-time experiments (72–168 h) targeting the 0:6 composition specifically are identified as a priority for future work (Section 4.6) to resolve the origin of this behavior.

Overall, the obtained results demonstrate that the sorption of [Au(CN)_2_]^−^ ions is a complex multistep process involving both diffusion and ion-exchange interactions. Although intraparticle diffusion contributes to the sorption mechanism, the excellent agreement with the PSO model confirms that specific interactions between gold cyanide complexes and the functional groups of the interpolymer system represent the dominant sorption mechanism. Among the investigated compositions, the TC007(Na^+^):AV-17-8(Cl^−^) system with a molar ratio of 1:5 exhibited the most efficient sorption kinetics.

The sorption kinetics of [Fe(CN)_6_]^4−^ ions were analyzed using the pseudo-first-order (PFO), pseudo-second-order (PSO), and Weber–Morris models (Table 4) to gain further insight into the selective sorption behavior of the investigated interpolymer systems. The PFO model provided a moderate fit to the experimental data, with R^2^ values ranging from 0.5144 to 0.9588. These results indicate that the sorption of ferrocyanide ions is not governed solely by external mass transfer or concentration gradients and that interactions with active sorption sites contribute significantly to the overall process.

According to the Weber–Morris model, intraparticle diffusion participates in the sorption mechanism; however, the deviation of the plots from the origin indicates that intraparticle diffusion is not the only rate-controlling step. Therefore, the sorption process involves a combination of external diffusion, intraparticle diffusion, and interactions between ferrocyanide ions and the functional groups of the sorbent. Owing to its higher charge and stronger hydration shell, the [Fe(CN)_6_]^4−^ complex experiences greater diffusion resistance than the [Au(CN)_2_]^−^ complex.

Among the investigated models, the PSO model exhibited the best agreement with the experimental data. For the 4:2, 3:3, 2:4, and 1:5 interpolymer systems, the correlation coefficients ranged from 0.9927 to 0.9992, indicating that ion exchange interactions play a dominant role in the sorption process. The highest rate constant (k_2_ = 1.2483 g mg^−1^ h^−1^) was obtained for the 2:4 composition, while similarly high values were observed for the 1:5 system. These results suggest that the mutual activation of functional groups within the interpolymer systems enhances the interaction between ferrocyanide ions and active sorption sites.

In contrast, the individual AV-17-8(Cl^−^) resin exhibited a very low fit to the PSO model (R^2^ = 0.0207), highlighting the advantages of the interpolymer systems over the single anion exchanger. Comparison of the kinetic characteristics of gold and iron cyanide complexes revealed that the PSO model adequately describes the sorption of both species. However, [Au(CN)_2_]^−^ ions exhibited higher sorption efficiency and stronger affinity toward the active sites of the interpolymer systems. This behavior can be attributed to the lower charge and weaker hydration of the gold cyanide complex, which facilitate its transport and interaction with functional groups.

Overall, the obtained results demonstrate that the sorption of [Fe(CN)_6_]^4−^ ions is a multistep process involving both diffusion and ion-exchange interactions. Although ferrocyanide ions are effectively sorbed by the investigated systems, their sorption efficiency remains lower than that of [Au(CN)_2_]^−^ ions, thereby confirming the selective character of the TC007(Na^+^):AV-17-8(Cl^−^) interpolymer systems toward gold cyanide complexes.

The non-zero intercepts (C > 0) of all linear segments confirm that intraparticle diffusion is not the sole rate-limiting step, and that film diffusion at the external bead surface contributes to the overall kinetic resistance, particularly in the early stages of sorption. The intraparticle diffusion rate constant *k*_*id*_ for [Au(CN)_2_]^−^ exceeded that for [Fe(CN)_6_]^4−^ in the intermediate diffusion stage, consistent with the faster transport of the smaller, singly charged linear dicyanoaurate anion through the polymer gel network relative to the larger, tetra-charged octahedral ferrocyanide complex.

### 3.4. Selectivity Analysis

#### 3.4.1. Selectivity Coefficients as a Function of IPS Composition

The selectivity coefficient β = D_Au_/D_Fe_, where *D* = *q*_*e*_/*C*_*e*_, was calculated for each IPS composition at equilibrium (48 h). The results, presented in Table 5, demonstrate a clear maximum at the 1:5 ratio (β = 4.95), confirming this composition as the selectivity optimum within the investigated range.

The physical interpretation of this maximum is as follows: at very high AV-17-8 fractions (approaching 0:6), both ions are sorbed with near-equal high efficiency, driving β toward unity. As the cation-exchanger fraction increases, the interfacial electrostatic repulsion selectively suppresses [Fe(CN)_6_]^4−^ uptake more than [Au(CN)_2_]^−^ uptake due to the greater charge of the ferrocyanide anion, thereby increasing β. At very high cation-exchanger fractions, the total anion-exchange capacity of the IPS becomes insufficient to sorb either ion meaningfully, and β becomes unreliable due to the near-zero sorption of both. The 1:5 ratio represents the optimal balance between sufficient anion-exchange capacity and the electrostatic selectivity effect imposed by the sulfonate cation-exchange component.

For the investigated interpolymer systems, increasing the TC007(Na^+^):AV-17-8(Cl^−^) molar ratio from 5:1 to 1:5 resulted in a gradual increase in the distribution coefficient of [Au(CN)_2_]^−^ ions from 0.0301 to 0.3062. Over the same range, the distribution coefficient of [Fe(CN)_6_]^4−^ ions also increased from 0.0178 to 0.0618. The selectivity coefficient (β) was found to be 1.69, 2.00, 2.01, 2.66, 4.95, and 1.67 for the 5:1, 4:2, 3:3, 2:4, 1:5, and 0:6 molar ratios, respectively (Table 5). The highest selectivity was observed at a molar ratio of 1:5, indicating that the interpolymer system exhibited a significantly greater affinity toward [Au(CN)_2_]^−^ ions than toward [Fe(CN)_6_]^4−^ ions under these conditions.

#### 3.4.2. Desorption Performance

Desorption kinetics from the loaded IPS were studied using 9% thiourea + 2% H_2_SO_4_ solution as the eluent over 72 h. At the 1:5 ratio, the desorbed mass of Au reached 22.74 mg/L at 72 h, corresponding to a desorption efficiency of approximately 98.5% relative to the total sorbed gold mass. The desorbed Fe mass was 17.05 mg/L at the same time point. This differential desorption behavior (Au desorbing more completely than Fe) further supports the conclusion that gold cyanide interacts with the IPS through a more reversible ion-exchange mechanism, whereas ferrocyanide may form partially irreversible electrostatic interactions with multiple exchange sites simultaneously due to its higher charge. The near-complete recovery of gold by 9% thiourea + 2% H_2_SO_4_ elution confirms the practical regeneration ability of the IPS system, which is a critical requirement for any hydrometallurgical sorbent. These results demonstrate regeneration ability over a single sorption–desorption cycle. Multi-cycle regeneration experiments, tracking Au and Fe extraction degree and selectivity coefficient β over successive cycles, are required to establish long-term operational stability and are identified as a priority for future work (Section 4.6); the present data should not be interpreted as evidence of stable performance over extended reuse.

### 3.5. Proposed Sorption Mechanism

Based on the combined kinetic, spectroscopic, thermal, and selectivity data, the following sorption mechanism is proposed for the TC007(Na^+^):AV-17-8(Cl^−^) IPS at the optimal 1:5 ratio.

External film diffusion: Both [Au(CN)_2_]^−^ and [Fe(CN)_6_]^4−^ migrate from the bulk solution through the external liquid film to the IPS bead surface, driven by the concentration gradient. This stage is rate-limiting at very short contact times (< 30 min), as indicated by the steep initial slope of the Weber–Morris plot.

Intraparticle diffusion: The anions diffuse through the hydrated gel-phase network of the PS-DVB beads. The smaller, singly charged [Au(CN)_2_]^−^ (molecular dimensions: ~2.5 Å × 7.4 Å, linear geometry) diffuses more rapidly through the polymer network than the bulkier [Fe(CN)_6_]^4−^ (octahedral, ~8.6 Å diameter), as reflected in the higher *kid* value for Au in the intermediate diffusion stage.

Ion exchange at quaternary ammonium site: Both anions undergo ion exchange with Cl^−^ at the –N^+^(CH_3_)_3_ functional groups of AV-17-8. The driving force is electrostatic attraction between the cationic site and the anionic complex. PSO kinetics confirm this as the dominant rate-determining step.

Electrostatic discrimination at the IPS interface: At the contact boundary between TC007 sulfonate groups (–SO_3_^−^, Na^+^ form) and AV-17-8 quaternary ammonium groups, a localized interfacial electric field is established. The highly charged [Fe(CN)_6_]^4−^ experiences significantly greater electrostatic repulsion from the –SO_3_^−^ environment than [Au(CN)_2_]^−^, effectively creating a charge-selective barrier that restricts ferrocyanide access to exchange sites near the cation-exchanger interface. This is the principal mechanism by which the IPS achieves selectivity over a pure anion exchanger.

Stabilization via coordination: FTIR evidence (C≡N stretching at 2034.1 and 2108.7 cm^−1^) confirms that the metal–cyanide complexes are not merely electrostatically held but become structurally stabilized within the polymer matrix through short-range coordination-type interactions between the CN ligand and the positively charged ammonium nitrogen, partially restricting their desorption at short elution times.

This proposed mechanism is consistent with all experimental observations: the PSO kinetic dominance, the multilinear Weber–Morris profile, the FTIR C≡N band shifts, the TGA-DSC thermal perturbation post-sorption, the composition-dependent selectivity maximum at 1:5, and the differential desorption behavior of Au versus Fe.

### 3.6. Langmuir Isothermal Model

The adsorption isotherms were determined using the Langmuir model, which assumes monolayer adsorption on a homogeneous surface with uniform binding sites and constant adsorption energy across all sites. According to this model, the amount of metal ions adsorbed onto the ion-exchange resin increases hyperbolically with the equilibrium concentration in the liquid phase, approaching a maximum saturation value [31]. This relationship can be described by the following linearized Equation (9):

(9) $$\frac{1}{Q_{e}}=\frac{1}{Q_{m}K_{L}C_{e}}+\frac{1}{Q_{m}}$$

where Q_e_ is the adsorption capacity adsorbed at equilibrium, Q_m_ is the maximum adsorption capacity (anions mg/g of resin), k_L_ is the Langmuir adsorption constant (L/mg), and C_e_ is the equilibrium concentration of the adsorbate (mg/L).

**Figure 10:**
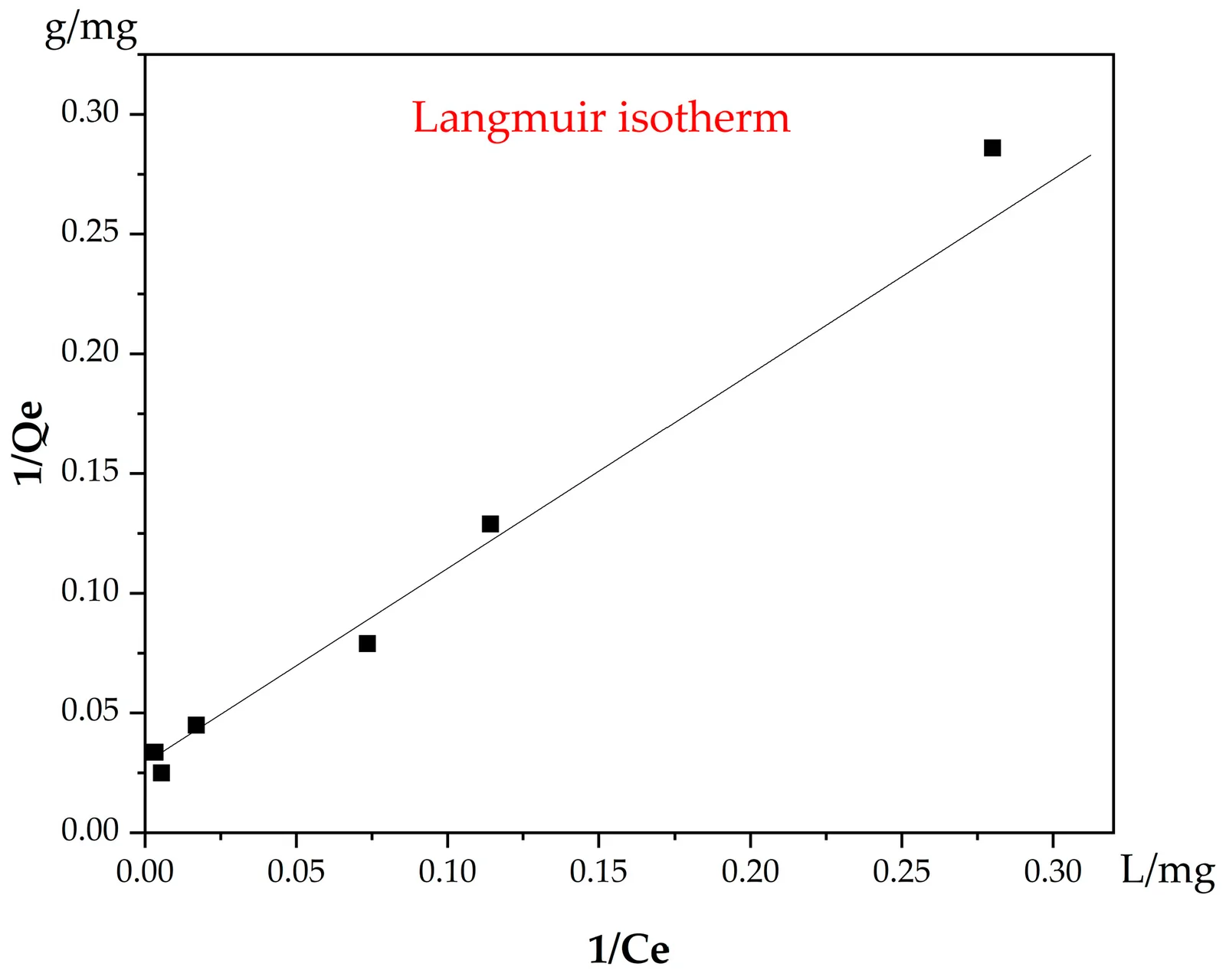
Langmuir isothermal model for the interpolymer system “TC007(Na^+^):AV-17-8(Cl^−^)” (1:5) Au(I).

The current work applies the Langmuir isotherm to characterize the adsorptive performance of the interpolymer system TC007(Na^+^):AV-17-8(Cl^−^) at a 1:5 molar ratio with respect to Au(I) ions, as illustrated in Figure 10. This sorbent configuration demonstrated a strong affinity for Au(I) uptake from solution, and its conformity to the Langmuir model—which assumes monolayer adsorption on a homogeneous surface with uniform binding sites and constant adsorption energy—confirms its suitability for targeted recovery applications.

Linear regression of the Langmuir isotherm (Figure 10) yielded a maximum adsorption capacity Q_m_ = 50.11 mg/g and a Langmuir constant K_L_ = 0.0212 L/mg, with a correlation coefficient of R^2^ = 0.9947, confirming excellent conformity to the Langmuir monolayer adsorption model for Au(I) uptake by the 1:5 IPS.

The standard Gibbs free energy of adsorption (ΔG°) was estimated from the Langmuir equilibrium constant using ΔG° = −RT ln(K°), where K° is the dimensionless thermodynamic equilibrium constant obtained by converting KL from L/mg to L/mol using the molar mass of Au (196.97 g/mol) and subsequently to a dimensionless form by multiplying by the standard-state molar concentration of water in dilute aqueous solution (55.5 mol/L), following established practice for avoiding the well-known unit-consistency problem in Langmuir-derived thermodynamic parameters. R is the universal gas constant (8.314 J mol^−1^ K^−1^) and T is the absolute temperature (298.15 K, matching the experimental conditions). This calculation yielded ΔG° = −30.62 kJ/mol, indicating a spontaneous and thermodynamically favorable adsorption process for Au(I) uptake by the TC007(Na^+^):AV-17-8(Cl^−^) (1:5) interpolymer system under the tested conditions. The magnitude of this value (in the range typically associated with chemisorption-dominated processes, generally −20 to −80 kJ/mol) is consistent with the pseudo-second-order kinetic behavior reported in Section 3.2 (R^2^ = 0.9992), which similarly indicates a chemisorptive, ion-exchange-dominated mechanism rather than simple physisorption.

Because the present isotherm data were collected at a single temperature (25 °C), the enthalpy (ΔH°) and entropy (ΔS°) of adsorption cannot be independently determined; their calculation requires Langmuir isotherms measured at multiple temperatures, analyzed via the Van’t Hoff equation (ln K° vs. 1/T). This is identified as a necessary extension for future work (Section 4.6) to fully characterize the thermodynamic driving forces of Au(I) uptake by the IPS.

## 4. Discussion

### 4.1. Novelty of the Interpolymer System Approach in Gold Cyanide Recovery

The central finding of this study—that a mixed-bed interpolymer system composed of a strong-acid cation exchanger and a strong-base anion exchanger achieves selective recovery of [Au(CN)_2_]^−^ over [Fe(CN)_6_]^4−^ with a selectivity coefficient β = 4.95 at the optimal 1:5 molar ratio—represents a meaningful advance over existing single-component ion-exchange approaches. Conventional strong-base anion exchangers, including industrially deployed resins of the quaternary ammonium type, sorb all anionic species present in solution without meaningful charge-density discrimination. The pure AV-17-8 system (0:6 ratio) reached only 37.55% Au and 26.45% Fe extraction at 48 h—markedly lower than every IPS composition from 2:4 through 1:5—while still exhibiting a low selectivity coefficient (β = 1.67), indicating that the presence of TC007 in the IPS compositions is associated with both higher absolute Au extraction and improved selectivity relative to the unmodified anion exchanger under the same contact times. This confirms that the anion exchanger alone is fundamentally incapable of separating [Au(CN)_2_]^−^ from the ferrocyanide matrix efficiently or selectively under the tested conditions.

The interpolymer system resolves this limitation not through a novel polymer architecture but through a compositionally tunable mixed-bed configuration in which the equivalent ratio of sulfonate to quaternary ammonium sites governs the electrostatic microenvironment experienced by competing anions. This distinguishes the approach conceptually from a simple physical blend used only to average bulk properties: the sorption and selectivity data (Section 3.1, Section 3.2, Section 3.3 and Section 3.4) show non-additive, ratio-dependent behavior (e.g., the ~30-fold PSO rate enhancement at 1:5 relative to pure AV-17-8) that cannot be predicted from the properties of either resin alone, indicating genuine inter-resin interaction rather than a rule-of-mixtures outcome. We do not claim novel network architecture, crosslinking design, or engineered interfacial morphology as sources of this behavior; these remain hypotheses requiring further characterization (Section 4.6).

### 4.2. Interpretation of Composition-Dependent Selectivity

The non-monotonic relationship between IPS molar ratio and selectivity coefficient β deserves careful interpretation. At low AV-17-8 fractions (5:1 ratio), the total anion-exchange capacity of the IPS is insufficient to sorb meaningful quantities of either ion, and selectivity is poorly defined. As the AV-17-8 fraction increases from 4:2 to 1:5, the anion-exchange capacity grows, and the concurrent presence of TC007 sulfonate groups begins to exert a differential inhibitory effect on the more highly charged ferrocyanide anion. The maximum at 1:5 represents the composition at which these two competing effects—increasing sorption capacity and increasing charge-selective discrimination—are optimally balanced.

This interpretation is supported by the charge asymmetry between the two competing anions. The [Au(CN)_2_]^−^ complex carries a single negative charge and has a linear molecular geometry with a relatively small cross-sectional area, whereas [Fe(CN)_6_]^4−^ carries a four-fold higher charge and adopts a bulky octahedral geometry with a hydrodynamic diameter that is approximately three times larger. In the vicinity of the –SO_3_^−^ groups of TC007, the Born electrostatic repulsion energy scales with the square of the ionic charge, meaning that [Fe(CN)_6_]^4−^ experiences repulsion that scales strongly with the square of ionic charge, favoring exclusion of the more highly charged [Fe(CN)_6_]^4−^ over [Au(CN)_2_]^−^, though the precise magnitude of this effect under the present ionic strength and hydration conditions has not been quantified experimentally. This charge-squared scaling provides a quantitative thermodynamic basis for the observed selectivity and predicts that IPS architectures will be inherently more effective for separating ions of widely differing charge than for separating isovalent anions—a useful design principle for future IPS development. This provides a generalized predictive rule: IPS selectivity will be most pronounced for ion pairs with the greatest charge asymmetry, and can, in principle, be tuned by adjusting the cation-exchanger fraction to match the charge differential of the target/competitor pair.

Beyond electrostatics, molecular size exclusion effects within the gel-phase PS-DVB matrix likely contribute to the observed selectivity. The gel-type structure of both TC007 and AV-17-8 imposes steric constraints on ion transport within the polymer network. The smaller, linear [Au(CN)_2_]^−^ anion is expected to diffuse more readily through the gel network than the bulky octahedral [Fe(CN)_6_]^4−^, consistent with the higher intraparticle diffusion rate constant observed for gold in the Weber–Morris analysis. This size-exclusion contribution, while secondary to the electrostatic effect, adds an additional layer of selectivity that is intrinsic to the gel-type IPS architecture and would not be present in macroporous or microporous exchanger variants.

It is important to note that the present dataset cannot uniquely isolate the proposed electrostatic-barrier mechanism from several plausible alternative or contributing explanations. First, simple dilution of the total anion-exchange capacity as the AV-17-8 fraction decreases would independently reduce sorption of both ions and could account for part of the reduced [Fe(CN)_6_]^4−^ uptake without invoking selective charge discrimination. Second, [Fe(CN)_6_]^4−^ possesses a larger hydrated radius and stronger hydration shell than [Au(CN)_2_]^−^ [cite hydration data], which would slow its diffusion independently of any electrostatic barrier. Third, bead-scale transport resistance—arising from the physical presence of TC007 beads interspersed among AV-17-8 beads—could sterically impede access to interior exchange sites for the larger ferrocyanide complex regardless of surface charge. Fourth, steric partitioning at the gel-phase pore scale, unrelated to electrostatics, could further disfavor the bulkier octahedral [Fe(CN)6]^4−^ ion. The present kinetic (Weber–Morris, PSO), FTIR, and TGA-DSC data are consistent with, but do not uniquely confirm, the electrostatic-barrier hypothesis, because none of these methods directly probes interfacial charge density or ion partitioning. Discriminating between these mechanisms would require zeta-potential or streaming-potential measurements of the IPS interface, ion-partition experiments at varying ionic strength (to test whether selectivity collapses under charge screening, as predicted by Donnan theory but not by pure steric/hydration mechanisms), or Poisson–Boltzmann modeling of the interfacial electrostatic potential. We have revised our conclusions (Section 5) to present the electrostatic-barrier concept explicitly as the leading working hypothesis rather than an established mechanism, pending such measurements.

### 4.3. Kinetic Mechanism and Comparison with Established Sorption Models

The universal applicability of the pseudo-second-order kinetic model across all six IPS compositions tested, with R^2^ values of 0.9984–0.9992, is a strong indicator of the chemisorptive, ion-exchange nature of the sorption process. The PSO model was originally derived under the assumption that the rate of sorption is proportional to the square of the number of unoccupied active sites, which is physically consistent with an ion-exchange mechanism in which two solution-phase anions compete for the same exchange site. Ho and McKay demonstrated [32] that PSO kinetics apply broadly to systems where chemisorption—defined as the formation of a strong electrostatic or coordinative bond between sorbate and sorbent—is the rate-controlling step, which aligns precisely with the ion-exchange interaction between [Au(CN)_2_]^−^ and the quaternary ammonium groups of AV-17-8 confirmed by FTIR.

The multilinear Weber–Morris profiles, with three identifiable linear segments and non-zero intercepts, are consistent with the well-established interpretation that ion exchange in polymeric gel-type sorbents proceeds through sequential film diffusion, intraparticle gel-phase diffusion, and equilibrium establishment stages. The non-zero intercept C > 0 across all segments confirms that intraparticle diffusion is not the sole rate-determining mechanism, and that external film resistance contributes, particularly at the earliest contact times. This finding has practical implications: under flow-through column conditions typical of CIP/CIL circuits, enhanced fluid velocity and turbulence would reduce film diffusion resistance and accelerate the approach to equilibrium, potentially improving gold recovery efficiency relative to the batch conditions investigated here.

Compared with kinetic data reported for gold cyanide sorption on conventional strong-base anion exchangers—where PSO rate constants typically fall in the range k_2_ = 0.01–0.05 g/(mg·h)—the IPS system exhibits rate constants that reflect the additional diffusion resistance introduced by the presence of the cation-exchange phase, which partially restricts ion transport. While this represents a kinetic trade-off relative to pure AV-17-8, the substantially improved selectivity (β = 4.95 vs. β = 1.67 for pure AV-17-8) justifies this trade-off for applications where gold–iron separation, rather than maximum extraction rate, is the primary objective.

### 4.4. FTIR and Thermal Evidence for the Sorption Mechanism

The FTIR spectroscopic evidence provides direct molecular-level confirmation of the proposed sorption mechanism. The appearance of C≡N stretching bands at 2108.7 and 2034.1 cm^−1^ in the post-sorption spectra of AV-17-8-containing IPS configurations is consistent with published assignments for metal cyanide complexes retained within ion-exchange matrices. The free [Au(CN)_2_]^−^ anion in aqueous solution exhibits a C≡N stretching frequency at approximately 2141 cm^−1^. The red shift observed upon sorption (Δν ≈ 32–107 cm^−1^) is attributable to a reduction in the C≡N bond order caused by the electrostatic interaction between the CN nitrogen lone pair and the positively charged quaternary ammonium nitrogen of the exchange site, effectively perturbing the triple-bond character of the cyanide ligand. This type of frequency perturbation has been reported for cyanide complexes interacting with nitrogen-donor functional groups in polymer matrices and provides spectroscopic support for the conclusion that the sorption involves more than simple electrostatic retention—a degree of coordinative interaction contributes to the stability of the sorbed complex.

The TGA-DSC data reinforce this interpretation. The appearance of a new thermal decomposition event in the 200–280 °C range in post-sorption IPS samples, absent in the virgin resins, is consistent with the thermal decomposition of retained metal–cyanide complexes. The endothermic character of this event in the DSC trace is indicative of bond-breaking processes requiring energy input, consistent with the decomposition of coordination compounds of moderate thermal stability. Importantly, this additional thermal event was observed across multiple IPS configurations—not only TC007:AV-17-8 but also KU-2-8:AV-17-8 and Lewatit:AV-17-8 systems—suggesting that metal–cyanide complex retention is a general property of anion-exchanger-containing IPS systems and is not sensitive to the specific identity of the cation-exchange partner. This generality strengthens the mechanistic argument and suggests that the IPS approach is broadly applicable to other cation/anion exchanger combinations.

### 4.5. Comparison with Published Studies

Relative to our previous KU-2-8/AV-17-8 IPS study and PMAA–P4VP hydrogel IPS work, the present study introduces (i) a different commercially scalable cation exchanger (TC007) with higher nominal capacity and distinct bead morphology, (ii) full compositional scanning across the 6:0–0:6 range under identical Au/Fe and pH conditions, and (iii) combined kinetic, spectroscopic, and thermal characterization of metal cyanide complex fixation. These elements were not previously reported for gold–iron separation in IPS systems and enable quantitative comparison of selectivity and kinetic enhancement across distinct IPS architectures.

The performance of the TC007(Na^+^):AV-17-8(Cl^−^) IPS at the 1:5 ratio compares favorably with several classes of sorbents reported in the literature for gold cyanide recovery. Activated carbon systems generally achieve high [Au(CN)_2_]^−^ recoveries (90–98%) but show limited discrimination against [Fe(CN)_6_]^4−^, with reported selectivity coefficients β_Au/Fe_ often below 2 under typical leach conditions [11], (p. 2256). Functionalized or chelating resins bearing sulfur- or nitrogen-donor ligands can reach β values of 8–12 in carefully controlled synthetic media [33], but these studies commonly employ lower Fe:Au ratios and lower ionic strengths than in industrial cyanide liquors. In this context, the β = 4.95 obtained for the 1:5 IPS in equimolar Au/Fe solutions sits between unmodified strong-base resins and specialized chelating materials, while relying solely on commercially available components and simple mechanical mixing.

Strong-base anion-exchange resins of the quaternary ammonium type, such as Purolite A500 and Lewatit MonoPlus M500, have been reported to achieve gold recovery efficiencies exceeding 95% but again with β values rarely exceeding 2–3 in iron-contaminated solutions [34]. The selectivity coefficient (β = 4.95) substantially exceeds that of the unmodified strong-base anion exchanger (β = 1.67) under identical equimolar conditions, though it remains below values reported for gold-specific chelating resins (β = 8–12); direct comparison is confounded by differing Fe:Au ratios and ionic strengths across studies.

Under the tested equimolar model solution conditions, the observed β = 4.95 is higher than that of the unmodified strong-base anion exchanger (β = 1.67) and falls within the lower part of the selectivity range reported for certain gold-specific functionalized resins, albeit under differing Fe:Au ratios and ionic strengths. Pseudo-second-order kinetic modeling provided the best fit to the experimental data (R^2^ = 0.9984–0.9992), indicating that ion-exchange interactions at quaternary ammonium sites play a dominant role in the overall sorption process, in agreement with FTIR and TGA-DSC observations.

### 4.6. Future Research Directions

The findings of this study open several productive avenues for future investigation:

Multi-temperature isotherm studies: Langmuir isotherms should be measured at multiple temperatures (e.g., 15–45 °C) to enable calculation of the enthalpy (ΔH°) and entropy (ΔS°) of Au(I) adsorption via the Van’t Hoff equation, providing a complete thermodynamic characterization of the driving forces for IPS-based gold recovery.

pH and ionic strength effects: The influence of solution pH (8–13) and competing electrolyte concentration on both sorption efficiency and selectivity should be systematically investigated, given the wide variation in leach solution chemistry across different ore types and processing routes.

Alternative IPS compositions: The IPS concept is not limited to the TC007/AV-17-8 pair. Macroporous variants of both exchangers, resins with different DVB crosslinking degrees, and IPS incorporating gold-selective chelating components represent logical next-generation materials.

Column dynamics and scale-up: Fixed-bed column experiments are essential for translating the batch selectivity results into practically applicable process parameters, including breakthrough capacity, elution profiles, and regeneration efficiency over multiple sorption–desorption cycles.

Multi-cycle regeneration testing: Sorption–desorption cycling (minimum 3–5 cycles) should be performed to quantify capacity and selectivity retention over repeated use, which is essential to substantiate claims of long-term operational stability for industrial deployment.

## 5. Conclusions

This study demonstrated for the first time that interpolymer systems (IPS) composed of a strong-acid sulfonated PS-DVB cation exchanger (TC007, Na^+^ form) and a strong-base quaternary ammonium PS-DVB anion exchanger (AV-17-8, Cl^−^ form) achieve selective recovery of dicyanoaurate(I), [Au(CN)_2_]^−^, over hexacyanoferrate(II), [Fe(CN)_6_]^4−^, from binary model cyanide solutions, with a performance that cannot be replicated by either component individually. The principal conclusions of the work are as follows.

IPS composition is the decisive determinant of both sorption efficiency and gold–iron selectivity. Among the seven molar ratios investigated (6:0 to 0:6), the 1:5 TC007(Na^+^):AV-17-8(Cl^−^) system delivered the optimal balance between anion-exchange capacity and charge-selective discrimination, achieving a maximum gold extraction degree of 79.88% and a selectivity coefficient β = 4.95 relative to ferrocyanide at 48 h equilibrium. Pure AV-17-8 (0:6) exhibited a markedly lower Au extraction degree (37.55%) than the 2:4 through 1:5 IPS compositions, following an anomalous, non-monotonic-rate kinetic profile, while its selectivity coefficient remained low (β = 1.67). These findings confirm that the cation-exchange component is essential not only for achieving charge-selective discrimination but also for promoting more complete Au uptake than the unmodified anion exchanger achieves alone under the tested contact times.

Sorption kinetics follow the pseudo-second-order model across all IPS compositions, with R^2^ values of 0.9984–0.9992, establishing ion exchange at the quaternary ammonium functional groups as the dominant rate-controlling mechanism. The pseudo-first-order model provided a significantly inferior fit, confirming the chemisorptive rather than physisorptive nature of the sorption process. Weber–Morris intraparticle diffusion analysis revealed a multilinear, three-stage kinetic profile with non-zero intercepts, indicating that both external film diffusion and intraparticle gel-phase diffusion contribute to the overall kinetic resistance, with practical equilibrium attained within 48 h.

The selectivity behavior is consistent with, but not uniquely proven by, charge-dependent electrostatic discrimination at the IPS interface. We propose as a working hypothesis that sulfonate groups of the cation-exchange component generate a residual anionic microenvironment that disproportionately repels the tetra-charged [Fe(CN)_6_]^4−^ relative to the singly charged [Au(CN)_2_]^−^, consistent with Born electrostatic theory in which repulsion energy scales with the square of ionic charge. Molecular size exclusion, differential hydration, and capacity dilution effects may also contribute and have not been experimentally isolated from the electrostatic contribution in the present study. Direct electrokinetic characterization (zeta/streaming potential) and ionic-strength-dependent selectivity experiments are required to confirm the relative magnitude of each contribution.

FTIR spectroscopy provided direct molecular-level confirmation of metal cyanide complex fixation within the IPS matrix. The appearance of C≡N stretching bands at 2108.7 and 2034.1 cm^−1^ in post-sorption spectra, representing a red shift relative to free [Au(CN)_2_]^−^ in solution (~2141 cm^−1^), indicates that the sorbed complexes are stabilized through both electrostatic ion-exchange interactions and short-range coordinative perturbation of the CN triple bond by the quaternary ammonium nitrogen of the exchange site.

TGA-DSC analysis confirmed the thermal incorporation of metal–cyanide complexes into the IPS structure, evidenced by a new endothermic decomposition event in the 200–280 °C range that was absent in virgin resins. This behavior was observed across multiple IPS configurations (TC007:AV-17-8, KU-2-8:AV-17-8, and Lewatit:AV-17-8) demonstrating that thermally stable metal–cyanide complex retention is a general feature of anion-exchanger-containing interpolymer systems.

Near-complete gold recovery (>98%) was achieved by 9% thiourea + 2% H_2_SO_4_ elution over 72 h, confirming the practical regeneration ability of the IPS system. The preferential desorption of gold over iron under 9% thiourea + 2% H_2_SO_4_ elution further supports the reversible ion-exchange character of the Au–IPS interaction and suggests that optimized two-stage elution protocols could yield high-purity gold fractions from complex ferrocyanide-containing leach solutions.

Critically, the ~30-fold kinetic rate enhancement (k_2_ = 0.8348 vs. 0.0273 g mg^−1^ h^−1^) observed for the optimal IPS relative to the pure anion exchanger reveals that the cation/anion exchanger interface does not merely impose steric and electrostatic barriers but actively promotes sorption site accessibility—a phenomenon we attribute to mutual functional group activation and which has no established analogue in single-component ion-exchange literature. This represents a newly identified kinetic benefit of the IPS architecture with broad implications for mixed-bed sorbent design.

Taken together, these findings establish the TC007(Na^+^):AV-17-8(Cl^−^) interpolymer system as a compositionally tunable, mechanistically well-characterized, and practically regenerable sorbent platform for selective gold recovery in hydrometallurgical processing. The use of commercially available, industrially scaled ion-exchange components—prepared by simple mechanical mixing without chemical modification—makes this approach immediately transferable to pilot-scale evaluation. Future work should prioritize pH and ionic strength effects, dynamic column experiments under realistic leach solution conditions, and DFT computational modeling of the interfacial charge-selective mechanism to provide a complete thermodynamic and quantum-chemical description of the IPS selectivity phenomenon. Because the present study employed equimolar [Au(CN)_2_]^−^/[Fe(CN)_6_]^4−^ model solutions without other cyanide species and evaluated sorption only under batch conditions at pH 10, the reported β and kinetic parameters should be viewed as baseline performance metrics rather than direct predictors of behavior in complex industrial liquors. Future work in dynamic columns and in multi-component synthetic or real leach solutions will be essential to validate the practical robustness of the IPS concept.

## Figures and Tables

**Table 1:** Dry masses of the polymers in the interpolymer system “TC007(Na^+^):AV-17-8(Cl^−^)”.

Components	6:0	5:1	4:2	3:3	2:4	1:5	0:6
m_TC007_ (g)	0.6155	0.4953	0.3965	0.2975	0.1981	0.0991	0.0000
m_AV-17-8_ (g)	0.0000	0.1095	0.2195	0.3295	0.4395	0.5492	0.6594
m_TC007(Na^+^):AV-17-8(Cl^−^)_ (g)	0.6155	0.6048	0.6160	0.6270	0.6376	0.6483	0.6594

**Table 2:** Surface elemental composition (wt.%) of TC007(Na^+^) and AV-17-8(Cl^−^) before and after sorption, determined by SEM-EDX.

Sample	C	N	O	Cl	Na	S	K	Ca	Fe	Au
TC007(Na^+^) (initial)	38.07	0.00	34.92	0.00	13.37	13.47	0.00	0.16	0.00	0.00
AV-17-8(Cl^−^) (initial)	78.69	0.00	0.00	21.31	0.00	0.00	0.00	0.00	0.00	0.00
TC007(Na^+^) (after sorption)	0.00	0.00	38.01	0.00	3.55	35.21	19.93	0.00	0.00	3.30
AV-17-8(Cl^−^) (after sorption)	54.96	18.26	6.78	0.35	0.00	0.00	0.00	0.00	6.1	13.55

**Table 3:** Parameters of kinetic models describing the sorption of [Au(CN)_2_]^−^ ions by TC007(Na^+^):AV-17-8(Cl^−^) interpolymer systems.

Kinetic Model	Parameters	Molar Ratios of TC007(Na^+^):AV-17-8(Cl^−^), [Au(CN)_2_]^−^					
		5:1	4:2	3:3	2:4	1:5	0:6
Pseudo-first-order	k_1_	0.0639	0.0677	0.0821	0.1526	0.1054	0.0199
	R_2_	0.5156	0.8499	0.6509	0.8223	0.6628	0.6919
Weber–Morris	b coefficient	1.3567	2.6378	3.1549	4.8446	6.2916	0.1419
	R^2^	0.5690	0.8206	0.7250	0.5562	0.4920	0.9038
Pseudo-second-order	k_2_	0.3359	0.3033	0.1644	0.6179	0.8348	0.0273
	R^2^	0.9984	0.9982	0.9968	0.9973	0.9992	0.7531

**Table 4:** Parameters of kinetic models describing the sorption of [Fe(CN)_6_]^4−^ ions by TC007(Na^+^):AV-17-8(Cl^−^) interpolymer system.

Kinetic Model	Parameters	Molar Ratios of TC007(Na^+^):AV-17-8(Cl^−^), [Fe(CN)_6_]^4−^					
		5:1	4:2	3:3	2:4	1:5	0:6
Pseudo-first-order	k_1_	0.0203	0.0301	0.0877	0.1176	0.1215	0.0143
	R^2^	0.5144	0.6273	0.6528	0.5989	0.9588	0.9576
Weber–Morris	b coefficient	0.2623	1.5788	2.0640	3.1658	3.3176	0.4386
	R^2^	0.8634	0.8536	0.6941	0.6314	0.8169	0.8344
Pseudo-second-order	k_2_	0.0892	0.2916	0.1929	1.2483	0.6852	0.0004
	R^2^	0.8816	0.9927	0.9981	0.9988	0.9992	0.0207

**Table 5:** Selectivity coefficients of TC007(Na^+^):AV-17-8(Cl^−^) interpolymer system toward [Au(CN)_2_]^−^ ions.

Molar Ratio	5:1	4:2	3:3	2:4	1:5	0:6
Distribution coefficient, [Au(CN)_2_]^−^	0.0301	0.0564	0.1104	0.1412	0.3062	0.0456
Distribution coefficient, [Fe(CN)_6_]^4−^	0.0178	0.0282	0.0549	0.0530	0.0618	0.0273
Selectivity coefficient (β)	1.69	2.00	2.01	2.66	4.95	1.67

## Data Availability

The original contributions presented in this study are included in the article. Further inquiries can be directed to the corresponding authors.

## Abbreviations

The following abbreviations are used in this manuscript:

IPS	Interpolymer systems
PS-DVB	Polystyrene–divinylbenzene copolymer
TC007	Commercial sulfonated PS-DVB cation-exchange resin (Lanlang^®^ TC007)
AV-17-8	Commercial quaternary ammonium PS-DVB anion-exchange resin AV-17-8
KU-2-8	Industrial cation-exchange resin KU-2-8
PMAA	Poly(methacrylic acid)
P4VP	Poly(4-vinylpyridine)
CIP	Carbon-in-pulp (activated-carbon process)
CIL	Carbon-in-leach (activated-carbon process)
ICP-OES	Inductively Coupled Plasma Optical Emission Spectrometry
FTIR	Fourier Transform Infrared spectroscopy
TGA-DSC	Combined Thermogravimetric Analysis–Differential Scanning Calorimetry
DTG	Derivative Thermogravimetry
AES	Atomic Emission Spectrometry
PFO	Pseudo-first-order kinetic model
PSO	Pseudo-second-order kinetic model
Weber–Morris	Weber–Morris intraparticle diffusion model
SEM	Scanning Electron Microscopy
EDX/EDS	Energy-Dispersive X-ray Analysis
DSC	Differential Scanning Calorimetry
